# Host-specialized fibrinogen-binding by a bacterial surface protein promotes biofilm formation and innate immune evasion

**DOI:** 10.1371/journal.ppat.1007816

**Published:** 2019-06-19

**Authors:** Amy C. Pickering, Pauline Vitry, Valeriia Prystopiuk, Brandon Garcia, Magnus Höök, Jeffrey Schoenebeck, Joan A. Geoghegan, Yves F. Dufrêne, J. Ross Fitzgerald

**Affiliations:** 1 The Roslin Institute and Edinburgh Infectious Diseases, University of Edinburgh, Easter Bush Campus, Edinburgh, Scotland, United Kingdom; 2 Institute of Life Sciences, Université Catholique de Louvain, Louvain-la-Neuve, Belgium; 3 Department of Microbiology and Immunology, Brody school of Medicine, East Carolina University, Greenville, North Carolina, United States of America; 4 Center for Infectious and Inflammatory Diseases, Texas A&M Health Science Center, Houston, Texas, United States of America; 5 The Roslin Institute and Royal (Dick) School of Veterinary Studies, University of Edinburgh, Easter Bush Campus, Edinburgh, Scotland, United Kingdom; 6 Department of Microbiology, Moyne Institute of Preventive Medicine, School of Genetics and Microbiology, Trinity College Dublin, Dublin, Ireland; 7 Walloon Excellence in Life Sciences and Biotechnology, Wavre, Belgium; New York University School of Medicine, UNITED STATES

## Abstract

Fibrinogen is an essential part of the blood coagulation cascade and a major component of the extracellular matrix in mammals. The interface between fibrinogen and bacterial pathogens is an important determinant of the outcome of infection. Here, we demonstrate that a canine host-restricted skin pathogen, *Staphylococcus pseudintermedius*, produces a cell wall-associated protein (SpsL) that has evolved the capacity for high strength binding to canine fibrinogen, with reduced binding to fibrinogen of other mammalian species including humans. Binding occurs via the surface-expressed N2N3 subdomains, of the SpsL A-domain, to multiple sites in the fibrinogen α-chain C-domain by a mechanism analogous to the classical dock, lock, and latch binding model. Host-specific binding is dependent on a tandem repeat region of the fibrinogen α-chain, a region highly divergent between mammals. Of note, we discovered that the tandem repeat region is also polymorphic in different canine breeds suggesting a potential influence on canine host susceptibility to *S*. *pseudintermedius* infection. Importantly, the strong host-specific fibrinogen-binding interaction of SpsL to canine fibrinogen is essential for bacterial aggregation and biofilm formation, and promotes resistance to neutrophil phagocytosis, suggesting a key role for the interaction during pathogenesis. Taken together, we have dissected a bacterial surface protein-ligand interaction resulting from the co-evolution of host and pathogen that promotes host-specific innate immune evasion and may contribute to its host-restricted ecology.

## Author summary

Many bacterial pathogens are specialized for a single host-species and rarely cause infections of other hosts. Our understanding of the bacterial factors underpinning host-specificity are limited. Here we demonstrate that a canine host-restricted bacterial pathogen, *Staphylococcus pseudintermedius*, produces a surface protein (SpsL) that has the ability to preferentially bind to canine fibrinogen with high strength. This host-specific interaction has evolved via binding to a tandem repeat region of the fibrinogen α-chain which is divergent among mammalian species. Importantly, we found that the strong binding interaction with canine fibrinogen promotes bacterial aggregation and biofilm formation as well as inhibiting neutrophil phagocytosis. Our findings reveal the host-adaptive evolution of a key bacterium-host interaction that promotes evasion of the host immune response.

## Introduction

Many bacteria evolve strict mutualistic relationships with their host species with limited capacity to colonize and cause disease in other hosts. In contrast, other bacteria have the ability to expand into new host-species leading to the emergence of new pathogenic clones. Our understanding of the bacterial and host factors that underpin pathogen-host ecology is very limited. However, bacterial surface proteins are central mediators of host colonization, and tissue tropism and, as such, are likely to play a critical role in determining host ecology [1, 2]. For example, the choline-binding protein A, of the major human pathogen *Streptococcus pneumoniae*, binds to polymeric immunoglobulin receptor, secretory component, secretory IgA, and factor H of complement from humans but not from other animal species tested [1]. In addition, the human host-restricted *Streptococccus pyogenes* expresses surface-anchored M protein that binds exclusively to human CD46 mediating binding and invasion of epithelial cells. Adaptive diversification of bacterial surface proteins can also have a major impact on tissue tropism and disease manifestation. For example, uropathogenic *Escherichia coli* virulence has arisen due to mutations in the fimbrial adhesin FimH, promoting high affinity binding to the urinary epithelium [3]. Similarly, a single non-synonymous mutation in a fibronectin-binding autolysin of *Staphylococcus saprophyticus*, associated with a selective sweep, has been linked to the pathogenesis of urinary tract infection in humans [4]. Additionally, single amino acid substitutions in the fibronectin-binding protein A (FnBPA) of *Staphylococcus aureus*, are associated with cardiac device infections and bacteremia in humans due to increased binding affinity for fibronectin [5–7].

Fibrinogen is a highly abundant protein in blood and is required for blood coagulation, thrombosis and host immune defense [8]. This large glycoprotein is composed of three chains, termed the α-, β-, and γ-chains, that form a dimer of trimers [8]. During coagulation, thrombin cleaves the fibrinogen α- and β-chains allowing fibrin formation, with the γ-chain binding directly to platelets to produce the blood clot [8]. Bacterial pathogens have evolved many mechanisms to bind to host fibrinogen to disrupt blood coagulation as well as promote host cell adherence, immune evasion and abscess formation [9, 10]. The importance of this interaction is highlighted by the large number of fibrinogen-binding proteins of bacteria that have been identified, with *S*. *aureus* encoding at least 9 fibrinogen-binding proteins [9–12]. It is unclear if each of these proteins confer an exclusive function via distinct fibrinogen-binding sites, or if convergent evolution is driving a high redundancy for fibrinogen-binding. In *S*. *aureus* there are fibrinogen-binding proteins that exhibit host-specificity and those that exhibit a broader host tropism. In the case of clumping factor B (ClfB) a host-restrictive fibrinogen-binding phenotype is observed due to the interaction with a sequence unique to the human fibrinogen α-chain [13]. Conversely, clumping factor A (ClfA) interacts with fibrinogen from multiple hosts, such as human, canine, and murine, due to an interaction with the fibrinogen γ-chain [14]. A single residue substitution of Q407A in the ovine fibrinogen γ-chain is sufficient to eliminate binding of ClfA to ovine fibrinogen [14]. As FnBPA adheres to the same region in the fibrinogen γ-chain, it is assumed that it exhibits the same host phenotype but this has not been directly investigated [15].

*Staphylococcus pseudintermedius* naturally colonizes the nares and perineum of healthy dogs and is a major cause of canine skin infections, particularly in canine breeds that are genetically pre-disposed to atopic dermatitis including boxers, German shepherds, golden retrievers, Dalmatians, Labrador retrievers, French bulldogs, West Highland white terriers, Jack Russell terriers, and shar-peis [16–19]. Although *S*. *pseudintermedius* occasionally causes zoonotic infections of humans via dog bite wounds, it is highly host-restricted and there is limited evidence for colonization or transmission among non-canine host species such as humans and cats [17]. Of note, *S*. *pseudintermedius* demonstrates a host-specific preference for corneocytes collected from healthy dogs in comparison to human healthy volunteers [20], suggesting the existence of bacterial surface factors that promote a canine host-tropism. However, the bacterial factors underpinning the canine host-restricted ecology of *S*. *pseudintermedius* are unknown. Previously, we identified a complement of 18 genes encoding cell wall-associated proteins of *S*. *pseudintermedius* strain ED99 and discovered 2 fibrinogen-binding proteins, SpsD and SpsL, which exhibited host-dependent variation in fibrinogen-binding after heterologous expression by *Lactococcus lactis* [21]. While SpsD was encoded by closely-related species of staphylococci associated with non-canine host-species, SpsL was specific for *S*. *pseudintermedius* [21] and was shown to be required for abscess formation in a murine skin infection model [22]. Previous sequence and structural analysis of SpsL identified a similar domain architecture to the fibronectin-binding proteins of *S*. *aureus* with an amino acid identity of ~27% [21]. In *S*. *pseudintermedius* strain ED99, SpsL is a protein of 930 amino acids that contains the typical signatures of a cell wall-associated protein with an N-terminal signal peptide and C-terminal LPKTG anchor motif [21]. The N-terminal of SpsL consists of an A-domain with N1, N2, and N3-subdomains that would be predicted to mediate fibrinogen-binding [21]. The C-terminal contains 7 tandem repeats that share 91–100% pairwise identity and 24% protein identity to the fibronectin-binding repeats of FnBPA [21]. These C-terminal repeats of SpsL have been demonstrated to confer fibronectin-binding that can mediate internalization of *S*. *pseudintermedius* into canine epithelial cells [23].

Here we dissect the interaction of SpsL with fibrinogen and demonstrate its functional consequences. Using atomic force microscopy (AFM) we quantify the enhanced binding force of SpsL for canine fibrinogen, which is dependent on a tandem repeat region in the fibrinogen α-chain that is genetically diverse in mammalian species. Further, we demonstrate that the strong host-specific interaction with canine fibrinogen is required for SpsL-mediated aggregation and biofilm formation, and promotes neutrophil opsonophagocytosis suggesting a key role for SpsL-fibrinogen binding in the pathogenesis and canine host ecology of *S*. *pseudintermedius*.

## Results

### SpsL expressed on the surface of *S*. *pseudintermedius* mediates host-specific binding to fibrinogen

**Fig 1:**
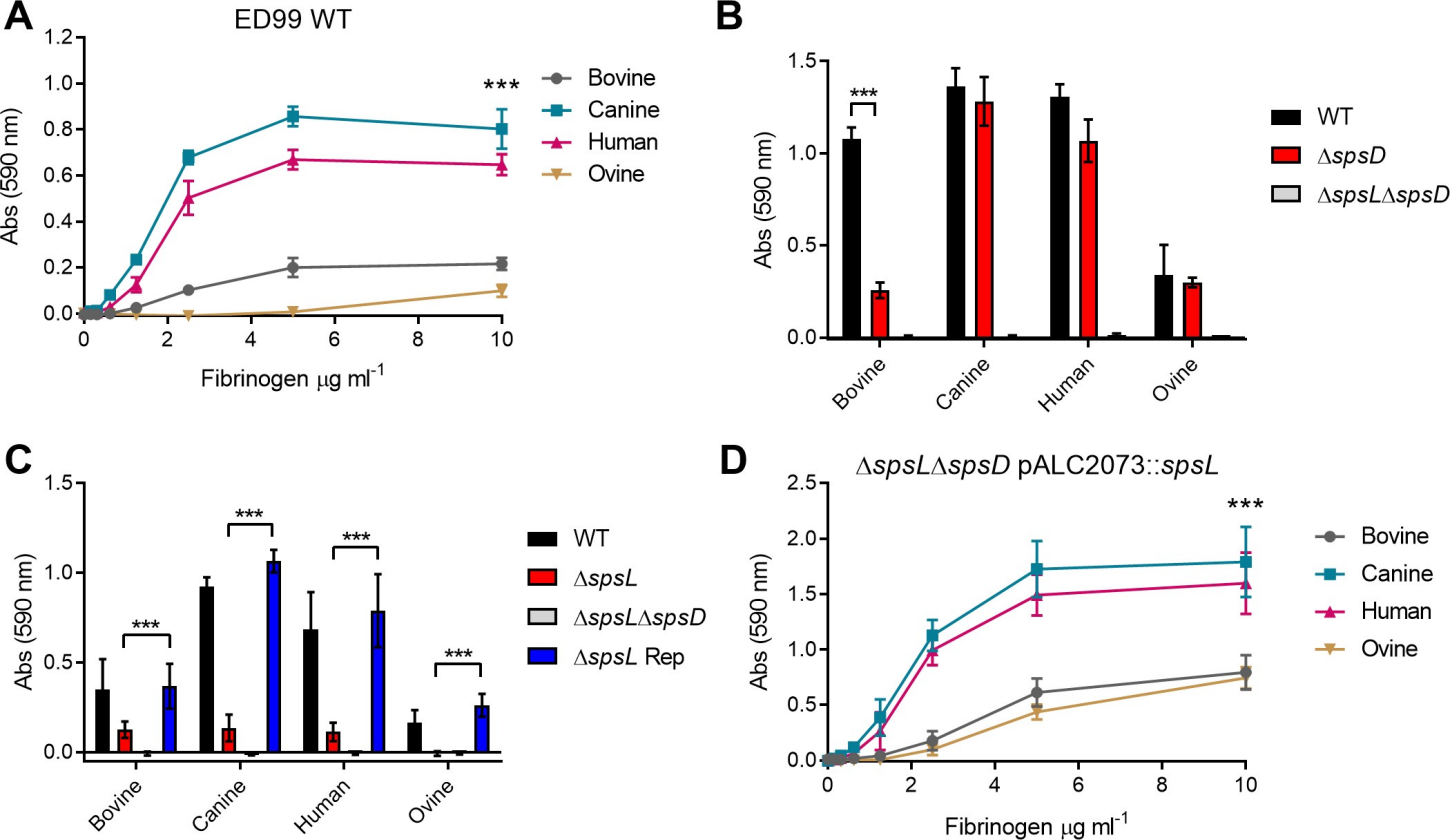
SpsL exhibits host-specific binding to fibrinogen. Bacterial adherence assays using crystal violet staining were used to quantify fibrinogen-binding from 4 host species–bovine, canine, human, and ovine. (A) Adherence of *S*. *pseudintermedius* ED99 wild type (WT) when cultured to mid-exponential growth phase. Data points represent the mean ± SD (n = 3). Differences in binding to fibrinogen was analyzed at 10 μg ml^-1^ fibrinogen (p ≤ 0.001, two-way ANOVA) (B) Adherence of WT, ED99Δ*spsD*, and ED99Δ*spsL*Δ*spsD* to 20 μg ml^-1^ fibrinogen when cultured to early-exponential growth phase. (C) Adherence of WT, ED99Δ*spsL*, ED99Δ*spsL*Δ*spsD*, and ED99Δ*spsL* Rep to 20 μg ml^-1^ fibrinogen when cultured to mid-exponential growth phase. Error bars represent SD (n = 9). Differences in fibrinogen-binding was analyzed (p ≤ 0.001, t-test) (D) Adherence of ED99Δ*spsL*Δ*spsD* expressing SpsL. Data points represent the mean ± SD (n = 9). Differences in binding to fibrinogen from multiple hosts was analyzed at 10 μg ml^-1^ (p ≤ 0.001, two-way ANOVA).

Previously, heterologous expression of SpsL in *L*. *lactis* revealed a host-dependent binding to fibrinogen [21]. In order to investigate this preliminary observation, we examined the capacity of wild-type *S*. *pseudintermedius* strain ED99 to bind to immobilized fibrinogen from different host species. At mid-exponential growth phase, the highest binding was observed to canine and human fibrinogen, with very limited binding to bovine and ovine fibrinogen (Fig 1A). A *S*. *pseudintermedius* ED99 mutant deficient in expression of a fibrinogen-binding protein SpsD (ED99Δ*spsD*), cultured to early-exponential growth phase, demonstrated binding to fibrinogen that was equivocal to wild-type ED99 for canine, ovine and human fibrinogen but reduced for bovine fibrinogen (p<0.001) (Fig 1B). In contrast, a mutant deficient in SpsL (ED99Δ*spsL*), cultured to mid-exponential growth phase, exhibited highly reduced binding to fibrinogen from all host species (p<0.001) (Fig 1C), with complete loss of fibrinogen-binding by a mutant deficient in both SpsL and SpsD (ED99Δ*spsL*Δ*spsD*), at both early-exponential (Fig 1B) and mid-exponential growth phases (Fig 1C). Re-introduction of the deleted *spsL* gene restored fibrinogen-binding as did complementation of ED99Δ*spsL*Δ*spsD* with a plasmid (pALC2073::*spsL*) encoding SpsL (Fig 1C and 1D). In summary, these data indicate that *S*. *pseudintermedius* ED99 has host-specific interactions with fibrinogen that are primarily mediated by SpsL. However, these adherence assays do not allow quantification of the strength of the binding interaction between SpsL and fibrinogen.

### SpsL demonstrates an enhanced binding strength for canine fibrinogen

**Fig 2:**
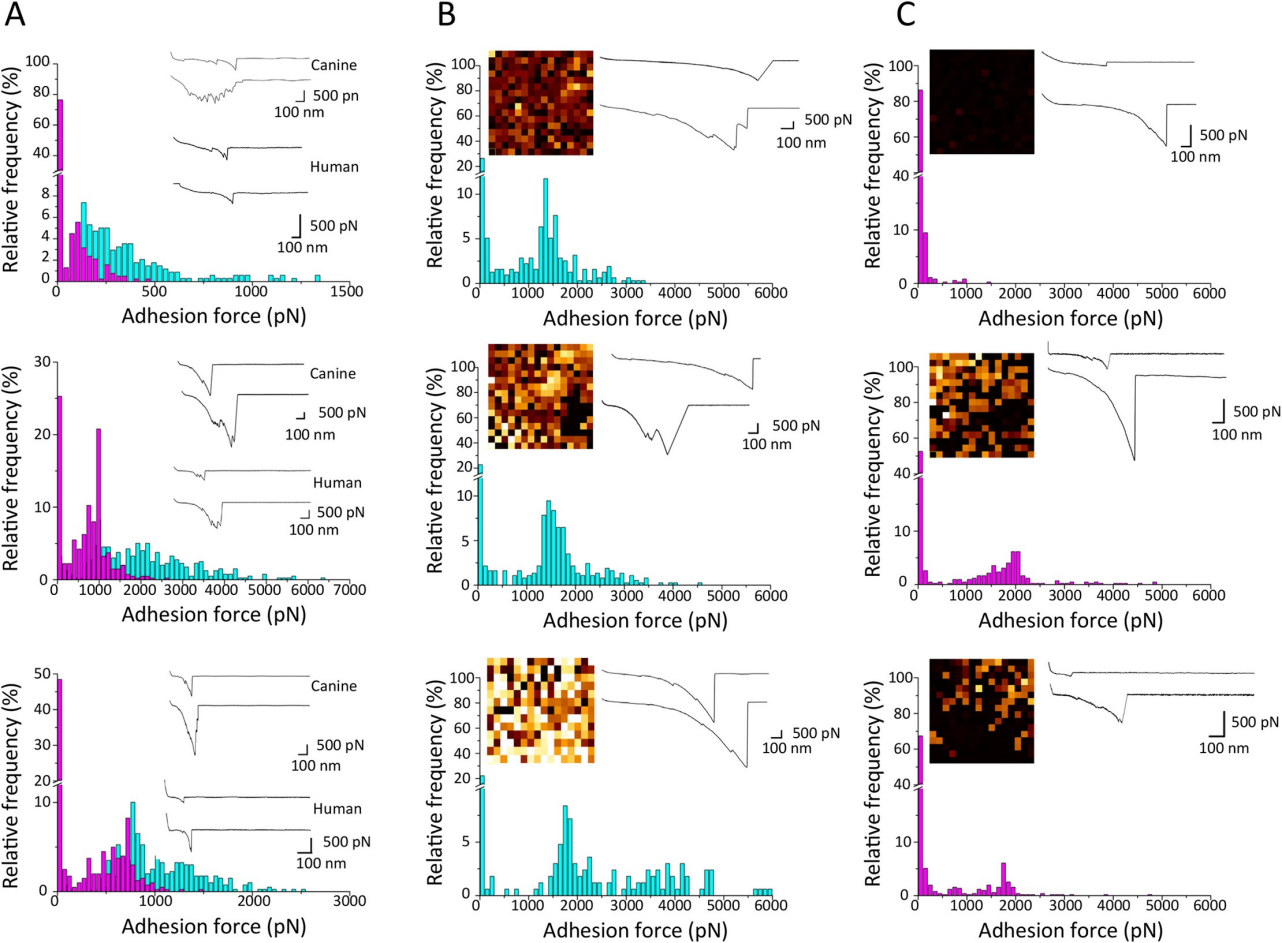
Atomic force microscopy analysis demonstrates increased binding strength of SpsL for canine fibrinogen. (A) Single-cell force spectroscopy (SCFS) measured the forces between single bacterial cells expressing SpsL A+SD and surfaces coated with either canine (blue) or human (purple) fibrinogen. Shown here are the adhesion force histograms with representative force curves obtained by recording force-distance curves in PBS for 3 different cells. All curves were obtained using a contact time of 100 ms, a maximum applied force of 250 pN, and approach and retraction speeds of 1,000 nm.s^-1^. (B, C) Single-molecule force spectroscopy (SMFS) captured the localization and binding strength of single adhesins on living bacteria. Adhesion force histograms obtained by recording force curves in PBS across the surface of single bacteria with either canine (B) or human (C) fibrinogen tips. The insets show adhesion force maps (scale bars: 100 nm, color scales: 3,000 pN) and representative force curves. Each bright pixel represents the detection of single proteins, no binding is represented by a black pixel and strong binding is represented by a white pixel. All curves were obtained using a contact time of 100 ms, a maximum applied force of 250 pN, and approach and retraction speeds of 1,000 nm.s^-1^.

In order to compare the molecular forces driving the binding of SpsL to canine and human fibrinogen, we used atomic force microscopy (AFM) [24, 25]. Firstly, for single-cell force spectroscopy (SCFS, Fig 2A), single bacteria were attached onto AFM cantilevers, and force-distance curves were collected between the cell probes and fibrinogen-coated surfaces (Fig 2A). The adhesion forces obtained for three representative cells interacting with either human or canine fibrinogen are presented (Fig 2A). While there was substantial variation between cells, the binding probability was always higher for canine rather than for human fibrinogen (85% *vs* 56%; means ± 12 and 28, from a total of n = 1,139 and 1,176 curves). Also, binding forces were stronger for canine fibrinogen (355 ± 354 pN from *n* = 228 adhesive curves; 2,077 ± 1,157 pN (n = 388), and 1,024 ± 427 pN (n = 352), for cell #1, cell #2 and cell #3, respectively), than for human fibrinogen (149 ± 84 pN (n = 85), 744±467 pN (n = 362), and 541±266 pN (n = 216)). Next, we used single-molecule force spectroscopy (SMFS) with fibrinogen-coated AFM tips to quantify the strength of single bonds (Fig 2B and 2C). Canine fibrinogen (Fig 2B) always showed very large forces (1,237 ± 754 pN from n = 258 adhesive curves; 1,554 ± 828 pN (n = 308), and 2,630 ± 1,393 pN (n = 137), for cell #1, cell #2 and cell #3, respectively). Of note, these high forces are in the range of values reported previously for the high-affinity “dock, lock and latch” binding of SdrG to fibrinogen [26]. For human fibrinogen, these strong forces were also observed but much less frequently (Fig 2C). Taken together these data are consistent with a high affinity dock, lock and latch-based mechanism for the binding of SpsL to canine fibrinogen.

### The N2N3 subdomains of SpsL expressed on the bacterial cell surface are required for fibrinogen-binding

To investigate the region of SpsL required for fibrinogen-binding, an array of recombinant truncates of SpsL were generated and purified from *E*. *coli* as described in the Supplemental Methods section (S1A Fig). From structural and functional studies of related staphylococcal surface proteins, we predicted that the N2N3 subdomains of SpsL would be sufficient for fibrinogen-binding [27–29]. However, in ELISA-like assays, none of the purified recombinant truncates of SpsL exhibited binding to fibrinogen, with all peptides tested demonstrating binding equivocal to the negative control (fibronectin-binding domain of SpsD) (S1B–S1D Fig). In contrast, fibronectin-binding could be detected in the SpsL recombinant protein construct containing a single fibronectin-binding repeat (S1E Fig) [23]. Similarly, full-length or truncated SpsL A-domain fragments expressed and purified from *S*. *pseudintermedius* ED99Δ*spsL*Δ*spsD* supernatant did not adhere to canine fibrinogen, in contrast to a positive control of recombinant SpsD N2N3 purified from *E*. *coli* (S1F Fig). However, heterologous overexpression of SpsL on the surface of a fibrinogen-binding deficient *S*. *aureus* strain (SH1000Δ*clfA*Δ*clfB*Δ*fnbA*Δ*fnbB*) [30] promoted high levels of adherence to canine fibrinogen (S1G Fig). Taken together, these data suggest that SpsL requires bacterial cell surface attachment to mediate fibrinogen-binding. Accordingly, subsequent experiments employed SpsL constructs expressed on the surface of *S*. *pseudintermedius* ED99.

**Fig 3:**
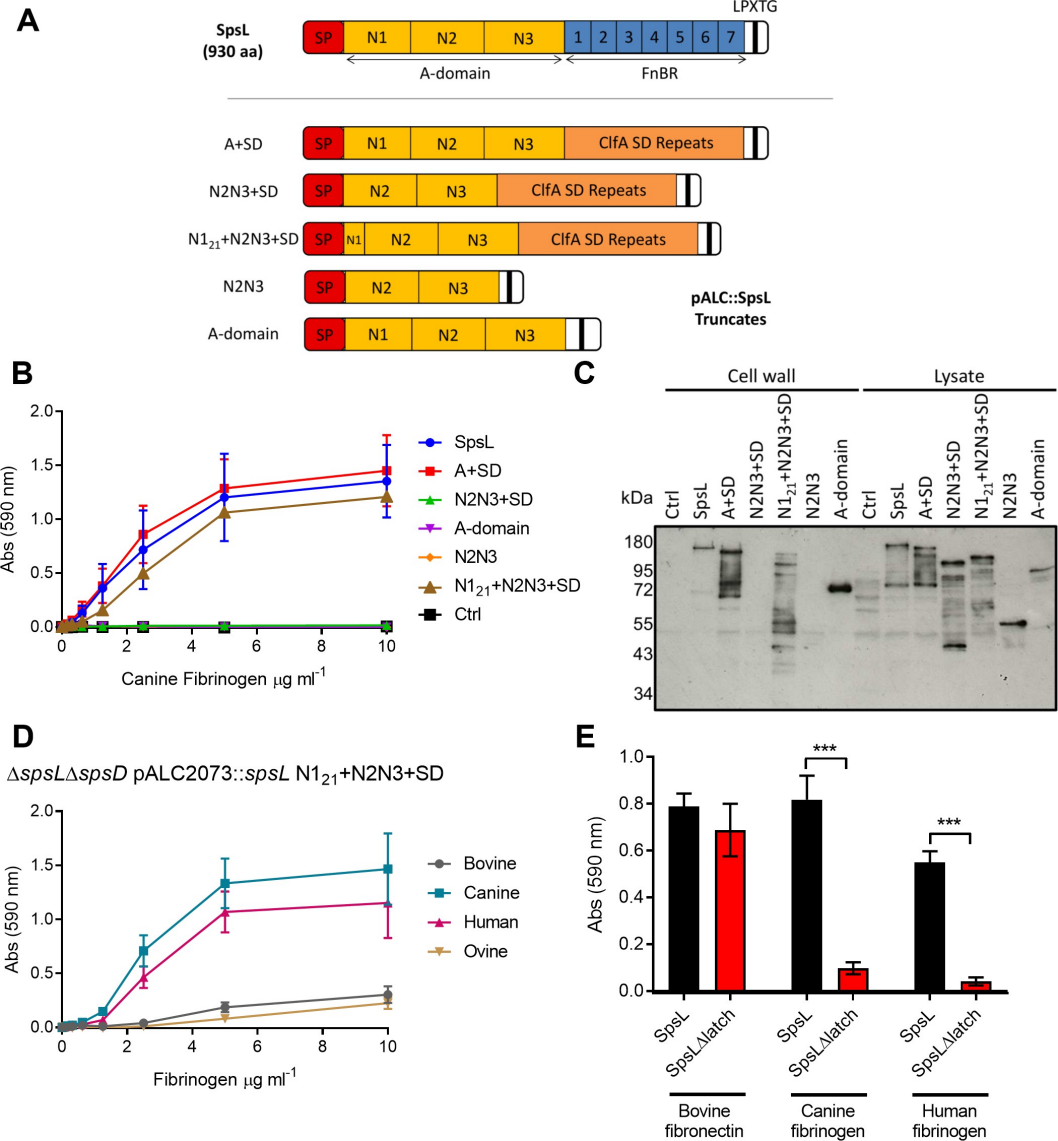
Surface-expressed SpsL N2N3 subdomains mediate host-specific fibrinogen-binding. (A) Schematic of the SpsL cell wall-associated truncates generated in this study. (B) Adherence of SpsL cell wall-associated truncates to canine fibrinogen using crystal violet staining. (C) Western blot analysis of cell wall-associated and cell lysate samples of each expression construct using chicken antibody generated against SpsL N2N3. (D) Adherence of SpsL N1_21_+N2N3+SD to fibrinogen from bovine, canine, human, and ovine hosts using crystal violet staining. Data points represent the mean ± SD (n = 9). (E) Adherence of SpsLΔlatch in comparison to full length SpsL to fibronectin, canine fibrinogen, and human fibrinogen coated at 20 μg ml^-1^ using crystal violet staining. The error bars represent SD (n = 9) (p ≤ 0.001, t-test).

SpsL fragments representing the A-domain and N2N3 subdomains were expressed on the surface of the *S*. *pseudintermedius* fibrinogen-binding deficient mutant ED99Δ*spsL*Δ*spsD* (Fig 3A). As reported for other bacterial cell wall-associated proteins, we considered that the C-terminal repeat region may be required to project the fibrinogen-binding domain from the cell surface, and that a small region of the N1 subdomain may be required for secretion and cell surface expression [31, 32]. To address this issue, chimeric proteins were generated that replace the SpsL fibronectin-binding repeats with the ClfA SD repeats (that do not exhibit any known ligand-binding activity) [33] and that contain a 21 amino acid region of the N1 subdomain (N1_21_; 181 VSKEENTQVMQSPQDVEQHVG 201) (Fig 3A). Analysis of the binding of these constructs to immobilized fibrinogen and expression analysis by Western blot indicated the requirement for the N1_21_ peptide for cell surface expression, with the N2N3+SD and N2N3 constructs not expressed on the cell surface (Fig 3B and 3C). Importantly, both the chimeric A-domain (A+SD) and N2N3-subdomain (N1_21_+N2N3+SD) proteins exhibited binding to canine fibrinogen that was equivocal to the full length SpsL protein (Fig 3B). The binding of the chimeric N1_21+_N2N3+SD protein to fibrinogen from multiple host species indicate that the SpsL N2N3 subdomains are sufficient for host-specific fibrinogen-binding, with the 21 amino acids of the N1 subdomain required for cell surface expression, (Fig 3D) suggesting that SpsL mediates ligand-binding in a manner analogous to the dock, lock and latch-binding mechanism described for other staphylococcal cell wall-associated proteins [34]. To investigate this further, we modelled the structure of SpsL N2N3 subdomains, based on the crystal structure of ClfA (pdb 1N67) [35]. The structural model predicted classical DE-variant IgG folds made up of β-sheets typical of staphylococcal fibrinogen-binding proteins (S2A Fig). From this model we identified a putative latch region, 502 NSASGSG 508, required for the dock, lock and latch binding mechanism (S2A Fig). Deletion of this putative latch region in a surface-expressed SpsL construct had no effect on surface expression or fibronectin-binding but abrogated adherence to both canine and human fibrinogen (p<0.001) (Fig 3E and S2B Fig). In addition to the AFM data, these results, suggest that the SpsL N2N3-subdomains expressed on the bacterial surface mediate fibrinogen-binding via a mechanism analogous to the dock, lock, and latch binding model.

### SpsL mediates enhanced binding to canine fibrinogen via a tandem repeat region of the fibrinogen α-chain

**Fig 4:**
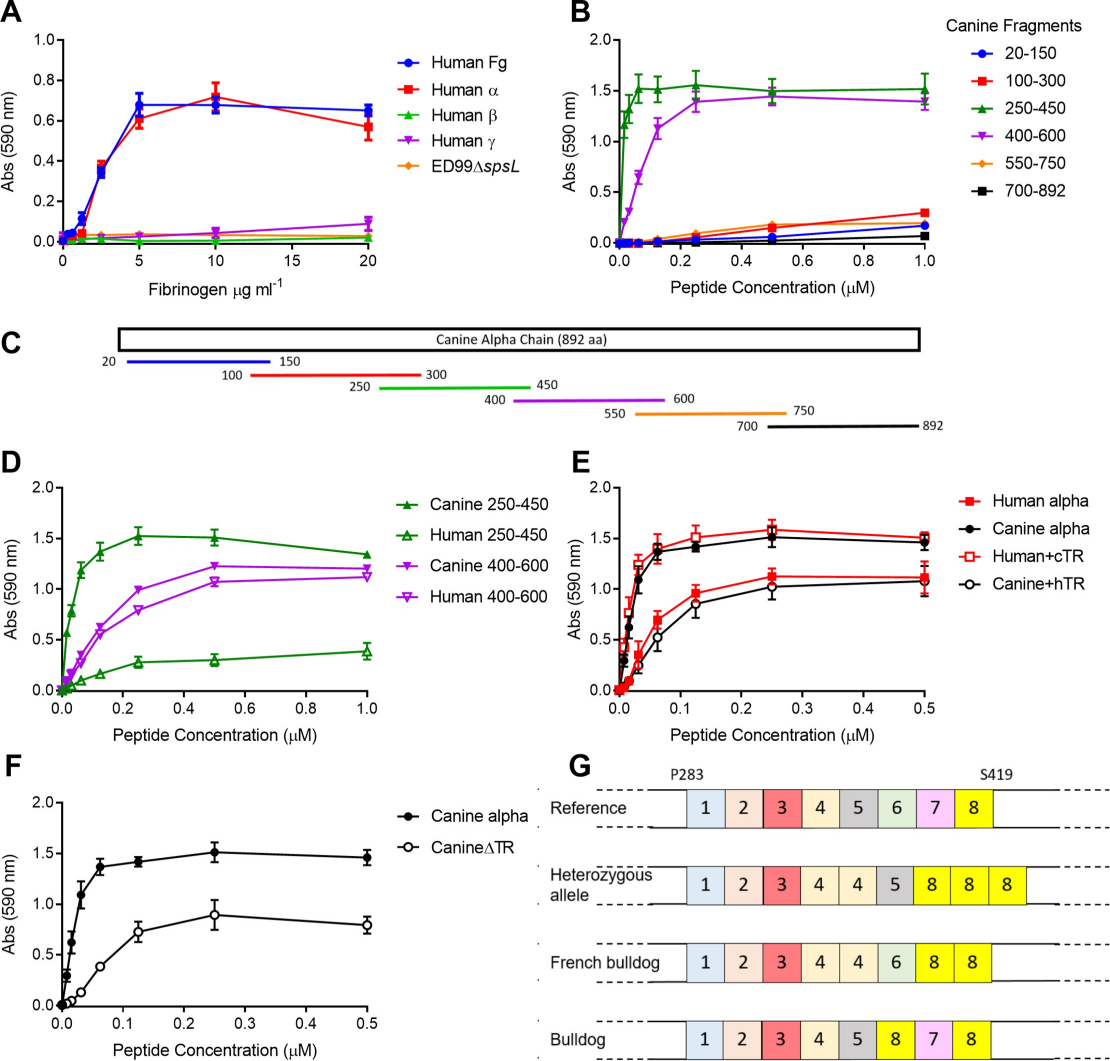
SpsL binds strongly to the tandem repeat region of the canine fibrinogen α-chain. (A) Adherence of ED99Δ*spsD* to human recombinant fibrinogen chains using crystal violet staining. (B, D, E, F) Adherence of SpsL expressed in ED99Δ*spsL*Δ*spsD* to fragments, chimeras, or deletion constructs of the canine and human fibrinogen α-chains. All data points represent the mean with error bars representing SEM (n = 9). (C) Schematic of the overlapping fragments generated in the canine α-chain. (G) Schematic of the diversity in the tandem repeat region of the canine fibrinogen α-chain in 11 canine breeds.

Staphylococcal proteins have evolved the ability to bind fibrinogen through multiple distinct interactions with different regions of host fibrinogen [13, 14, 36]. Previously it has been identified that *S*. *pseudintermedius* strain 326 is capable of binding to the fibrinogen α-chain with binding to the β-, and γ-chains not investigated [37]. To identify the binding site of SpsL, recombinant versions of the α-, β-, and γ-chains of human fibrinogen were expressed in and purified from *E*. *coli* and employed in bacterial binding assays. Both *S*. *pseudintermedius* ED99 and ED99Δ*spsD* demonstrated specific adherence to the human α-chain, but not to the β- or γ-chains revealing the α-chain as the receptor for SpsL binding (Fig 4A). To further refine the location of the SpsL binding site, 6 overlapping fragments of the canine fibrinogen α-chain were synthesized (NCBI reference sequence: XP_532697.2), purified from *E*. *coli*, and analyzed for adherence to ED99Δ*spsL*Δ*spsD* expressing full length SpsL (Fig 4B and 4C). SpsL demonstrated binding to two of the overlapping fragments (250–450 and 400–600 amino acids) that span the α-connector region of fibrinogen containing unordered tandem repeats (residues P283-S419) (Fig 4B). Purification of equivalent fragments derived from the human α-chain revealed equivocal binding to the 400–600 fragment but reduced binding to the human 250–450 fragment (Fig 4D). These data indicate that the canine fibrinogen α-chain contains strong (250–450) and weaker (400–600) SpsL binding sites while human fibrinogen contains just the weaker binding site (400–600). To confirm that the canine tandem repeat region is responsible for the host-specific interaction of SpsL with fibrinogen, we generated chimeric full-length proteins where the tandem repeat region from human and canine fibrinogen α-chains, respectively (P283-G421), were exchanged. The addition of the canine α-chain tandem repeats provides stronger binding to the human fibrinogen α-chain and in contrast the addition of the human α-chain tandem repeats provides weaker binding to the canine fibrinogen α-chain (Fig 4E). As a control, we examined the binding of ClfB expressed on the surface of SH1000Δ*clfA*Δ*clfB*Δ*fnbA*Δ*fnbB*. ClfB is a *S*. *aureus* fibrinogen-binding surface protein that binds specifically to repeat 5 of the human α-chain tandem repeats [13]. The host-specificity of ClfB was confirmed with specific binding observed to the human 250–450 fragment (S3A Fig), and the canine chimeric protein containing the human tandem repeat sequence (S3B Fig) but not to the canine 250–450 fragment or the canine alpha chain. These data demonstrate that the tandem repeat region of the fibrinogen α-chain is responsible for the host-specific interaction of SpsL.

The canine tandem repeat region of the fibrinogen α-chain contains 7 repeats of 18 amino acids and a partial repeat of 11 amino acids (S3C Fig) [38]. The generation of recombinant fragments spanning the tandem repeats of the fibrinogen α-chain (S3D Fig), revealed that SpsL is capable of binding to multiple regions in the canine tandem repeat region (S3E Fig). In addition, deletion of the whole tandem repeat region, in the canine α-chain, confirmed the presence of a weaker binding site (Fig 4F), which we localized to the region adjacent to the tandem repeats (S423-E474) in both canine and human fibrinogen (S3F and S3G Fig). Overall, these data demonstrate that SpsL mediates binding to multiple locations in the fibrinogen α-chain, and that the strong canine-specific interaction is dependent on the unique tandem repeat sequence present in the canine fibrinogen α-chain.

### The fibrinogen α-chain tandem repeat region exhibits canine breed-specific variation

The number of tandem repeats in the bovine fibrinogen α-chain have been reported to differ between cattle breeds [39]. To investigate if this is also the case for dogs we investigated publicly available canine sequences of the fibrinogen α-chain but, due to the repetitive nature of the tandem repeat region, the paired-end short sequence reads were not sufficient to support assembly and robust analysis. To overcome this, we isolated genomic DNA from 11 different canine breeds and PCR-amplified DNA specific for the P283-E474 region of the fibrinogen α-chain, followed by DNA sequencing. Sequence analysis, in comparison to NCBI reference sequence: XP_532697.2, revealed that the region of weaker binding (S423-E474) is conserved among the canine breeds examined (S4A Fig). In contrast, the French bulldog and Labrador retriever exhibited heterozygous alleles that contain an additional repeat unit in the stronger binding site (Fig 4G). This heterozygous allele, common to both breeds, contains a duplication of repeat 4 and amino acid substitutions that result in the replacement of repeats 6 and 7 with repeat 8 (XP_532697.2:p.[S347_S348insTRPGSTGPGSAGTWS;S373N;L394P]) (Fig 4G). The unique French bulldog allele contains substitutions that convert repeat 5 to repeat 4, and repeat 7 to repeat 8 (XP_532697.2:p.[S347_T351del;G352R;T361A;L394P]), with a unique bulldog allele replacing repeat 6 with repeat 8 (XP_532697.2:p.S373N) (Fig 4G). Overall, these analyses demonstrate that the canine-specific binding site of SpsL in the tandem repeat region of the canine fibrinogen α-chain has undergone genetic diversification during the evolution of different breeds of dog.

### SpsL promotes bacterial aggregation and biofilm formation in a host-restricted manner

**Fig 5:**
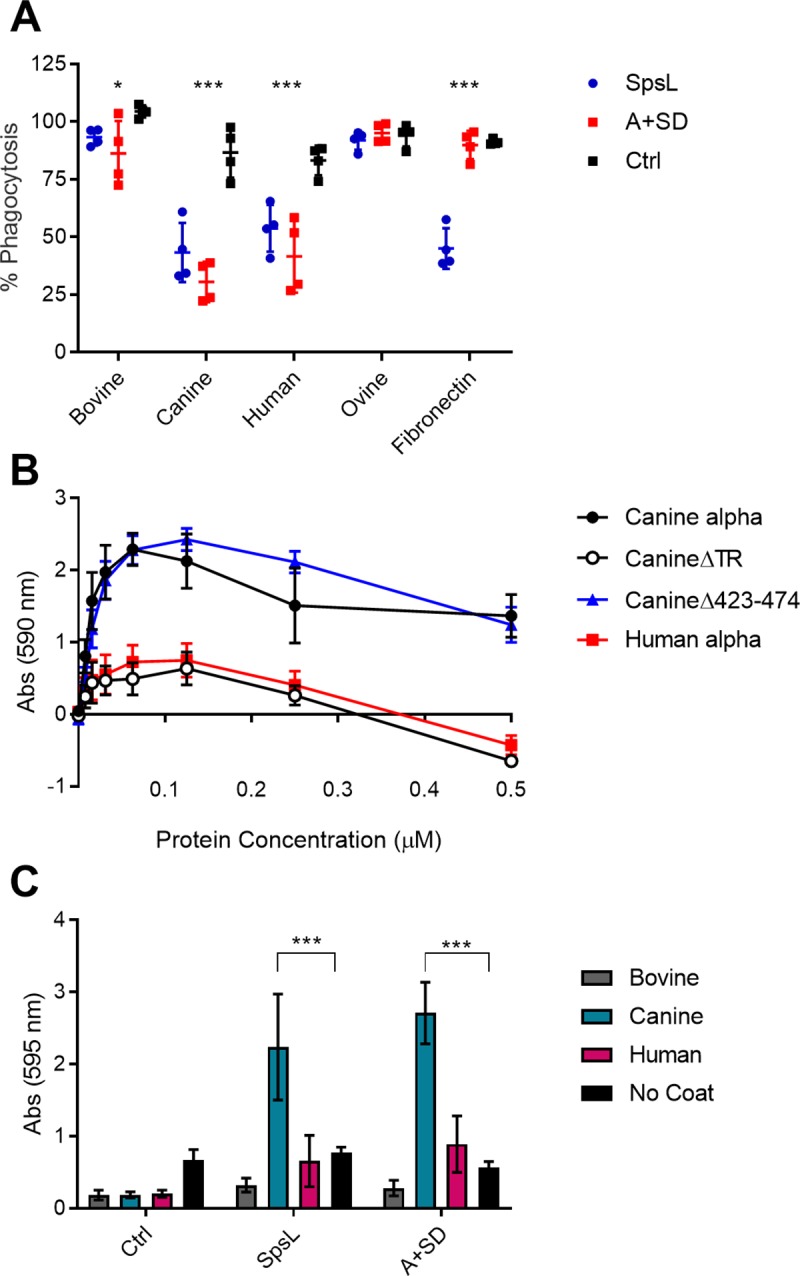
SpsL promotes evasion of neutrophil phagocytosis, bacterial aggregation and biofilm formation in a host-specific manner. (A) Differential phagocytosis of FITC-labelled bacteria expressing SpsL or A+SD opsonized with fibrinogen from bovine, canine, human, or ovine hosts or bovine fibronectin before being opsonized with 10% human serum. Data is presented as the percentage of cells containing phagocytosed bacteria in comparison to the serum-only control. Each data point represents the level of phagocytosis from neutrophils isolated from a single donor. Ctrl refers to bacteria containing an empty plasmid. The bar represents the mean and the error bars represent SD. Differences in phagocytosis were analyzed between all three bacterial groups for each opsonin (p ≤ 0.05 or p ≤ 0.001, one-way ANOVA). (B) Adherence of SpsL to canine fibrinogen α-chain in the presence of soluble purified peptides. Data points represent the mean ± SD (n = 9). (C) Biofilm formation by bacteria expressing SpsL or A+SD on surfaces coated with bovine, canine, or human fibrinogen. Error bars represent SD (p ≤ 0.001, one-way ANOVA) (n = 4).

The evolution of a strong canine-specific fibrinogen-binding interaction for SpsL suggests an important role in canine host-pathogen interactions. Accordingly, we investigated the impact of the interaction on phenotypes relevant to pathogenesis. Firstly, we considered if the interaction could promote inhibition of opsonophagocytosis as reported previously for fibrinogen-binding proteins of *S*. *aureus* ClfA and Efb [40, 41]. Accordingly, FITC-labelled bacteria were opsonized with bovine, canine, human, or ovine fibrinogen or bovine fibronectin and analyzed for phagocytosis by human neutrophils. As expected, full length SpsL, but not the chimeric A-domain protein (A+SD), inhibited phagocytosis in the presence of fibronectin (p<0.001) (Fig 5A). However, the ability of SpsL to inhibit neutrophil phagocytosis in the presence of fibrinogen was demonstrated to be host-specific with opsonophagocytosis inhibited in the presence of canine and human fibrinogen (p<0.001) but not in the presence of bovine or ovine fibrinogen (Fig 5A).

We next examined the role of SpsL-canine fibrinogen-binding on *S*. *pseudintermedius* aggregation and biofilm formation. The aggregation of *S*. *aureus* has been demonstrated to be important for the development of bloodstream infections [42, 43] and catheter-related infections [44]. In particular, it has been reported that fibrinogen-dependent *S*. *aureus* aggregation can stimulate the activation of virulence through a quorum-sensing dependent mechanism [45]. In order to examine the potential role of the canine-specific fibrinogen-binding in *S*. *pseudintermedius* aggregation, we attempted to block binding of *S*. *pseudintermedius* to the canine fibrinogen α-chain by including soluble fibrinogen in a bacterial adherence assay. Instead of blocking adherence, we found that soluble canine fibrinogen α-chain, but not human fibrinogen α-chain, supported the formation of surface bound aggregates (Fig 5B). Deletion of the weaker binding site (S423-E474) in the canine fibrinogen α-chain, had no effect on bacterial aggregation (Fig 5B). However, deletion of the stronger binding site (the tandem repeat region), resulted in complete abrogation of bacterial aggregation (Fig 5B). This demonstrates that SpsL promotes surface bound bacterial aggregation in a host-restricted manner. To further investigate the impact of fibrinogen on the aggregation of *S*. *pseudintermedius* we performed static biofilm assays in the presence or absence of fibrinogen from different host species (Fig 5C). Coating with canine fibrinogen supported enhanced biofilm formation among bacterial cells expressing SpsL than wells coated with either human or bovine fibrinogen demonstrating that the strong interaction of SpsL with canine fibrinogen promotes the initial attachment stage of biofilm formation (Fig 5C). Overall, these data demonstrate that the high strength interaction of SpsL with canine fibrinogen promotes bacterial aggregation and biofilm formation.

**Fig 6:**
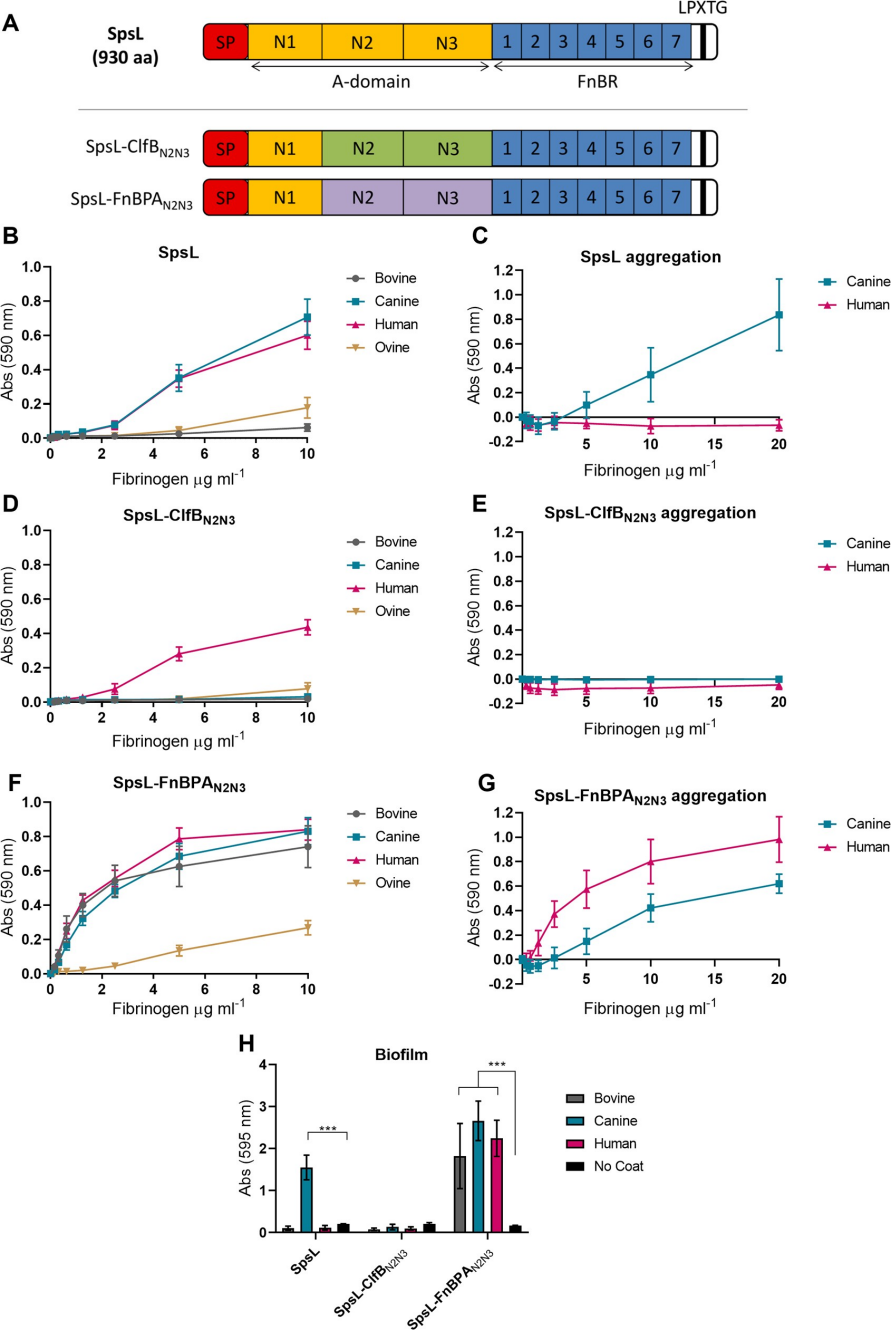
SpsL promotes bacterial aggregation and biofilm formation in a host-restricted manner. (A) Schematic of the SpsL chimeric cell wall-associated truncates generated. Bacterial adherence assays of full length SpsL (B), chimeric SpsL-ClfB_N2N3_ (D), or chimeric SpsL-FnBPA_N2N3_ (F) to fibrinogen from bovine, canine, human, and ovine. Data points represent the mean ± SD (n = 9). Adherence of full length SpsL (C), chimeric SpsL-ClfB_N2N3_ (E), or chimeric SpsL-FnBPA_N2N3_ (G) to canine fibrinogen or human fibrinogen in the presence of soluble canine or human fibrinogen. Data points represent the mean ± SD (n = 9). (H) Biofilm formation by bacteria expressing SpsL, chimeric SpsL-ClfB_N2N3,_ or chimeric SpsL-FnBPA_N2N3_ on surfaces coated with bovine, canine, or human fibrinogen. Error bars represent SD (p ≤ 0.001, one-way ANOVA) (n = 3).

In order to investigate if other staphylococcal fibrinogen-binding proteins exhibit similar host-specificity, we generated constructs expressing chimeric SpsL proteins that contain the fibrinogen-binding N2N3 subdomains of ClfB or FnBPA but maintain the SpsL promoter, signal peptide, fibronectin-binding repeats, and cell wall anchor (Fig 6A). The generation of chimeric proteins was favored over the expression of native ClfB or FnBPA proteins to limit variation in cell surface expression. The N2N3 subdomains of these proteins were selected because of the host-restriction of ClfB to human fibrinogen α-chain and the similar domain architecture of SpsL and FnBPA. As expected from our previous analysis, SpsL showed similar binding to both canine and human fibrinogen (Fig 6B). However, the high strength interaction of SpsL with canine fibrinogen is essential for bacterial aggregation (Fig 6C) and biofilm formation (Fig 6H). SpsL-ClfB_N2N3_ demonstrated a similar binding curve to SpsL but exhibited specific binding to human fibrinogen (Fig 6D) as previously predicted [13]. This human fibrinogen-binding was not sufficient to mediate bacterial aggregation (Fig 6E) or biofilm formation (Fig 6H) demonstrating that not all staphylococcal fibrinogen-binding proteins are capable of mediating these infection-related phenotypes. In contrast, SpsL-FnBPA_N2N3_ exhibited a binding pattern predicted from an interaction with the fibrinogen γ-chain with equivocal binding to bovine, canine, and human fibrinogen and reduced ovine fibrinogen-binding (Fig 6F). SpsL-FnBPA_N2N3_ is capable of mediating bacterial aggregation (Fig 6G) and biofilm formation (Fig 6H) in the presence of fibrinogen from all hosts tested suggesting that FnBPA does not have a host-restricted tropism. From this comparative analysis we can conclude that SpsL is unique in promoting bacterial aggregation and biofilm formation in a manner that corresponds to the host-restricted ecology of *S*. *pseudintermedius*.

## Discussion

The factors underpinning bacterial host-tropism are not well understood but often involve surface proteins mediating interactions with host cells and the extracellular matrix [1]. The genus *Staphylococcus* includes species such as *S*. *aureus* which have a multi-host tropism with the capacity to switch between different host species. In contrast, some species such as *S*. *pseudintermedius* are highly host-restricted and although *S*. *pseudintermedius* can occasionally cause zoonotic infections of humans (typically through dog bite wounds), the capacity to spread in human populations has not been reported. The bacterial factors underpinning the host-restricted ecology of *S*. *pseudintermedius* are unknown. Previously, we demonstrated that SpsL contributed to abscess formation in a murine model of subcutaneous infection indicating that it is a virulence factor during the pathogenesis of skin infection [22]. The poor binding of SpsL to murine fibrinogen suggests that this effect is not mediated by the interaction of SpsL with murine fibrinogen [22]. Here we demonstrate that SpsL mediates high strength binding to canine fibrinogen in a host-specific manner and that this host-adaptation conferred the ability to mediate bacterial aggregation and biofilm formation. The role of SpsL-fibrinogen binding in canine pathogenesis cannot be formally tested *in vivo* by experimental infections of dogs in the UK due to ethical constraints. However, our *ex vivo* binding and cellular infection data reveal multiple pathogenic traits that depend on the host-specific interaction of SpsL and canine fibrinogen, suggesting a key role in the host ecology of *S*. *pseudintermedius*.

Cell surface proteins of *S*. *aureus* have been reported to contribute to tissue or disease tropism in humans. For example, the fibrinogen- and loricrin-binding protein, ClfB, exhibits greater adherence to skin corneocytes taken from atopic dermatitis patients with low levels of natural moisturizing factor suggesting a role in niche adaptation [46, 47]. ClfB interacts with the human tandem repeat region of the fibrinogen α-chain [13] but unlike SpsL, ClfB binds to a single site; namely repeat unit 5, and exclusively binds to human fibrinogen [13]. In addition to ClfB, the bone sialoprotein-binding protein (Bbp) and the extracellular fibrinogen-binding protein (Efb) also bind to the fibrinogen α-chain via distinct RGD-integrin-binding sites inhibiting thrombin-induced coagulation and platelet aggregation, respectively [36, 48]. In contrast, SpsL interacts with multiple sites in the canine fibrinogen α-chain; namely within the tandem repeats and their flanking regions, (Fig 4). Similarly, the serine-rich repeat glycoproteins of *Streptococcus agalactiae*, Srr1 and Srr2, bind to repeat units 6, 7, and 8 of the tandem repeat region of the human fibrinogen α-chain via a variation of the dock, lock, and latch binding mechanism, with Srr2 displaying a stronger binding affinity than Srr1 [49]. The enhanced binding affinity of Srr2 was linked with increased adherence to endothelial cells, which may be important for Group B *Streptococcus*-associated meningitis [49]. The ability of the Srr and SpsL proteins to adhere to more than one site in the tandem repeat region of the fibrinogen α-chain may have evolved as a mechanism for overcoming extant genetic diversity in this region between individuals within a host species as observed in the current study for SpsL (Fig 4G) [39, 50].

We were unable to detect binding of soluble SpsL proteins to canine fibrinogen by ELISA suggesting that immobilization and surface presentation is essential for SpsL functionality, even when full length SpsL is expressed as a recombinant protein (S1 Fig). To address this, we utilized AFM, demonstrating that bacterial surface-associated SpsL binds to fibrinogen via extremely strong binding forces (around 2000 pN) that are in the range of the strength measured for the dock, lock and latch interaction between fibrinogen and the structurally-related SdrG and ClfA [26, 51]. Dock, lock and latch forces have been shown to originate from hydrogen bonds between the ligand peptide backbone and the adhesin [52, 53], and are activated by mechanical tension, as observed with catch bonds [54]. Of note, ClfB has much greater affinity for loricrin when expressed on the bacterial cell surface rather than as a recombinant protein with the C-terminal stalk enhancing binding affinity [25]. A similar mechanism may be required for SpsL adherence to fibrinogen with the C-terminal repeat domain enhancing the ligand-binding affinity of the N2N3 subdomains. It is increasingly being recognized that analysis of protein-protein interactions on the bacterial cell surface is more physiologically relevant than testing the interaction of recombinant polypeptides [25, 55].

Our data reveal that the high strength canine-specific binding of SpsL facilitates several virulence phenotypes not previously reported for *S*. *pseudintermedius* including surface-bound bacterial aggregation. When *S*. *aureus* forms fibrinogen-dependent aggregates, *agr*-mediated quorum sensing is activated leading to the up-regulation of virulence gene expression [45]. Consequently, the inhibition of *S*. *aureus* aggregation *in vivo* has been linked with decreases in mortality from sepsis and protection from lethal lung injury [43, 56]. We also discovered that SpsL facilitates fibrinogen-dependent biofilm formation, a phenotype not previously reported for *S*. *pseudintermedius*. Such fibrinogen-dependent biofilms are observed in *S*. *aureus* strains isolated from skin infections [57], a phenomenon implicated in indwelling medical device infections [58]. In this regard, inhibition of fibrin formation reduced the development of *S*. *aureus* biofilms in a murine catheter infection model [58], and molecules targeting SpsL could be beneficial in preventing canine indwelling device infections caused by *S*. *pseudintermedius*. Finally, we have demonstrated that SpsL binding to soluble fibrinogen inhibits neutrophil phagocytosis, suggesting a role for SpsL in innate immune evasion. Taken together, we have dissected the host-dependent binding of a bacterial surface protein and demonstrated its importance for multiple pathogenic traits, providing new insights into the host-specific ecology of a major bacterial pathogen.

## Materials and methods

### Ethics statement

Chicken immunization was performed using unembryonated hen’s eggs at the Scottish national blood transfusion service (Pentland Science Park, Midlothian, UK). The procedures performed were carried out under the authority of the UK Home Office Project License PPL 60/4165 and Animals (Scientific Procedures) Act 1986 regulations.

Human venous blood was taken from consenting adult healthy volunteers in accordance with a human subject protocol approved by the national research ethics service (NRES) committee London City and East under the research ethics committee reference 13/LO/1537. Passive volunteer recruitment was conducted at the Roslin Institute (University of Edinburgh). Written consent was taken from each volunteer before blood collection and after an outline of the risks was provided. All blood collection samples were anonymized.

### Bacterial strains and culture conditions

The bacterial strains and plasmids used in this study are listed in S1 Table. *S*. *pseudintermedius* and *S*. *aureus* strains were routinely cultured in Brain Heart Infusion broth at 37°C with shaking and supplemented with 10 μg ml^-1^ chloramphenicol as required. *E*. *coli* strains were cultured in Luria broth at 37°C with shaking supplemented with 100 μg ml^-1^ ampicillin, 15 μg ml^-1^ tetracycline, or 25 μg ml^-1^ kanamycin as required.

### Source of extracellular matrix proteins

Fibrinogen isolated from bovine, human, and ovine plasma (Sigma-Aldrich) and bovine fibronectin (EMD Millipore) was sourced commercially. Canine fibrinogen was purified from Beagle sodium citrate whole blood (Lampire Biological Products) using a previously described method [59]. All fibrinogen samples were purified to remove contaminating fibronectin using Gelatin-Sepharose 4B (GE Healthcare). Depletion of fibronectin was confirmed by Western blot analysis using 1 μg ml^-1^ rabbit anti-fibronectin IgG (abcam) and 0.2 μg ml^-1^ goat anti-rabbit IgG-HRP (abcam).

### Bacterial adherence assay

Solid phase adherence assays were performed using *S*. *pseudintermedius* and *S*. *aureus* strains expressing pALC2073 or pCU1 constructs cultured to an OD_600nm_ of 0.6 and induced for protein expression with 3 μg ml^-1^ anhydrotetracycline for 2 h. Cells were washed and suspended in PBS to OD_600nm_ of 1.0. Wells were coated overnight at 4°C with fibrinogen from multiple hosts or recombinant α-chain fragments in a 96-well MaxiSorp plate (Nunc). After blocking with 8% (w/v) milk-PBS, bacteria were applied to the wells for 2 h at 37°C. After washing, adherent cells were fixed with 25% (v/v) formaldehyde (Sigma) for 30 min and stained with 0.5% (v/v) crystal violet (Sigma) for 3 min. The cell-associated stain was solubilized with 5% acetic acid (v/v) and analyzed using a Synergy HT plate reader (BioTek) at 590 nm wavelength.

For aggregation experiments, the same procedure was followed as stated above with the addition of either soluble fibrinogen or recombinant fibrinogen α-chain to the bacteria using two-fold serial dilution and then incubation for 2 h at 37°C.

### Cloning of expression constructs

The primers used in this study are listed in S2 Table. Initial expression constructs of the human and canine fibrinogen α-chains were synthesized by Integrated Design Technologies (IDT) using the DNA sequence of a female Boxer (NCBI reference sequence: XP_532697.2) as highlighted in S1 Table. For typical restriction-ligation cloning procedures, the region of interest was amplified (*PfuUltra* II Fusion HS Polymerase—Agilent) and blunt cloned into pSC-B using the StrataClone Blunt PCR Cloning Kit (Agilent). Restriction digestion of the plasmid of interest (pQE30, pT7, or pALC2073) and the blunt cloned PCR product was performed at 37°C for at least 2 h and purified using the Monarch Gel Extraction Kit (NEB). All digested plasmids were treated with Antarctic Phosphatase (NEB) before overnight ligation with T4 DNA Ligase (NEB) at a 3:1 molar ratio of insert:plasmid. Dialysis of the 20 μl ligation reactions was performed using 0.025 μm filter circular discs (Millipore) before electroporation into the appropriate *E*. *coli* strain–DC10B [60], DH5α (Invitrogen), or XL-1 Blue (Agilent). All plasmid constructs were verified by Sanger sequencing (Edinburgh Genomics, University of Edinburgh) before transformation into *E*. *coli* BL21 DE3 (Invitrogen), or appropriate *S*. *pseudintermedius* strain.

Some expression constructs were also produced using sequence ligase independent cloning (SLIC) as described previously [61]. Briefly, primers were designed to amplify the gene of interest as well as sequence complementation to the expression plasmid. Primers were also designed to amplify the plasmid of interest, pQE30, pALC2073 or pCT using Platinum PCR Supermix (Invitrogen) or *PfuUltra* II Fusion HS Polymerase (Agilent). All PCR products were purified using Monarch PCR & DNA Cleanup kit or Monarch Gel Extraction kit (NEB). T4 DNA Polymerase (NEB) was used to generate DNA overhangs on both the insert and plasmid PCRs with step-wise temperature increments used to anneal the complementary DNA sequences. The heat annealed constructs were electro-transformed into *E*. *coli* DC10B [60] or DH5α (Invitrogen) and verified using Sanger sequencing (Edinburgh Genomics, University of Edinburgh).

### Preparation of *Staphylococcus* competent cells and electro-transformation

*S*. *pseudintermedius* and *S*. *aureus* competent cells were produced using a method outlined previously [60]. Plasmids for electroporation were concentrated to 1 μg μl^-1^ using Pellet Paint co-precipitant (Novagen) and 5 μg used for the electro-transformation as previously described [23].

### Recombinant protein induction and purification

Recombinant hexa-Histidine-tagged proteins expressed in *E*. *coli* were cultured to OD_600nm_ of 0.6 and induced using 1 mM IPTG at either 37°C for 4 h or 16°C overnight. Recombinant α-chain proteins were purified under denaturing conditions (8M urea, 100 mM monosodium phosphate, 10 mM Tris-HCl) using Ni-NTA agarose (Invitrogen) and gravity flow columns (Bio-Rad). Bacterial lysis was performed in pH 8.0 binding buffer at room temperature with tilting for at least 1 h. Lysates were pelleted at 16000 x *g* for 20 min and the supernatant filter sterilized. Lysates were tilted at room temperature with conditioned Ni-NTA agarose for 1 h. The column was washed with pH 6.3 wash buffer and the protein eluted with pH 4.5 elution buffer. After analysis by 4–20% Mini-PROTEAN TGX precast gel (Bio-Rad), protein quantification was performed using a BCA assay (Novagen).

### Release of surface proteins from *S*. *pseudintermedius*

*S*. *pseudintermedius* cells were cultured to exponential phase (OD_600nm_ of 0.4–0.6). Cells were washed with PBS and suspended in lysis buffer (50 mM Tris-HCl, 20 mM MgCl_2_, pH 7.5) supplemented with 30% (w/v) raffinose and cOmplete protease inhibitor (Roche). Cell wall proteins were solubilized by incubation with 400 μg ml^-1^ lysostaphin at 37°C for 20 min. Supernatant samples were collected after protoplast recovery by centrifugation at 6000 x *g* for 20 min. The production of cell lysate samples was generated by lysing cell pellets in PBS on the One-Shot cell disruptor (Constant Systems) with 2 passes at 40 Kpsi.

### Generation of anti-SpsL N2N3 IgY Antibody

Recombinant His-tag SpsL N2N3 protein was used as antigen for chicken immunization and antibody generation at the Scottish national blood transfusion service (Pentland Science Park). The Eggspress IgY purification kit (Gallus Immunotech) was used to purify antibody from egg yolk. Further purification of the antibody was performed using CNBr-activated Sepharose 4B (GE Healthcare). This antibody was used in Western blot analysis to detect the expression of SpsL using 1 μg ml^-1^ chicken anti-SpsL N2N3 IgY and 0.5 μg ml^-1^ F(ab’)2 rabbit anti-chicken IgG-HRP (Bethyl Laboratories).

### Atomic force microscopy

#### Functionalization of substrates with fibrinogen

Gold-coated glass coverslips and cantilevers (OMCL-TR4; Olympus; nominal spring constant, ~0.02N m^-1^) were immersed overnight in an ethanol solution containing 1 mM 10% 16-mercaptododecahexanoic acid—90% 1-mercapto-1-undecanol (Sigma), rinsed with ethanol, and dried with N_2_. Substrates and cantilevers were then immersed for 30 min in a solution containing 10 mg ml^-1^ NHS and 25 mg ml^-1^ 1-ethyl-3-(3-dimethylaminopropyl)-carbodiimide (Sigma), rinsed with ultrapure water (ELGA LabWater), incubated with 0.1 mg ml^-1^ of fibrinogen for 1 h, rinsed further with PBS buffer, and immediately used without de-wetting.

#### Functionalization of cantilevers with fibrinogen

Functionalized tips were obtained using PEG-benzaldehyde linkers [62]. Prior to functionalization, cantilevers were washed with chloroform and ethanol, placed in a UV-ozone cleaner for 30 min, immersed overnight in an ethanolamine solution (3.3 g ethanolamine–6 ml dimethyl sulfoxide [DMSO]), and then washed 3 times with DMSO and 2 times with ethanol and dried with N_2_. The ethanolamine-coated cantilevers were immersed for 2 h in a solution prepared by mixing 1 mg Acetal–PEG–NHS dissolved in 0.5 ml of chloroform with 10 μl triethylamine and then washed with chloroform and dried with N_2_. Cantilevers were further immersed for 5 min in a 1% citric acid solution, washed in Ultrapure water (ELGA LabWater), and then covered with a 200 μl droplet of PBS solution containing 200 μg ml^-1^ of the fibrinogen to which 2 μl of a 1 M NaCNBH_3_ solution was added. After 50 min, cantilevers were incubated with 5 μl of a 1 M ethanolamine solution in order to passivate unreacted aldehyde groups and then washed with and stored in buffer.

#### Single-cell force spectroscopy

For all atomic force microscopy (AFM) experiments, cells expressing chimeric SpsL A+SD were harvested after overnight incubation, washed in PBS, and diluted 1:100 in PBS. For SCFS, bacterial cell probes were obtained as previously described [63, 64]. Briefly, colloidal probes were obtained by attaching a single silica microsphere (6.1 mm diameter; Bangs Laboratories) with a thin layer of UV-curable glue (NOA 63; Norland Edmund Optics) to triangle-shaped tipless cantilevers (NP-O10; Bruker) with a NanoWizard III AFM (JPK Instruments). The cantilevers were then immersed for 1 h in Tris-buffered saline (TBS; 50 mM Tris, 150 mM NaCl, pH 8.5) containing 4 mg ml^-1^ dopamine hydrochloride (Sigma), rinsed in TBS, and used directly for cell probe preparation. The nominal spring constant of the colloidal probe was determined by the thermal noise method. 50 μL of diluted bacterial suspension was deposited into a glass Petri dish containing fibrinogen (human and canine) coated substrates at a distinct location within the Petri dish, and 3 ml of PBS was added to the system. The colloidal probe was brought into contact with an isolated bacterium and retracted to attach the bacterial cell; proper attachment of the cell on the colloidal probe was checked using optical microscopy. Cell probes were used to measure cell–substrate interaction forces at room temperature, using an applied force of 250 pN, a constant approach-retraction speed of 1 μm s^–1^, and a contact time of 0 ms. Data were analyzed using the Data Processing software from JPK Instruments. Adhesion force and distance rupture histograms were obtained by calculating the maximum adhesion force and rupture distance of the last peak for each curve.

#### Single-molecule force spectroscopy

For SMFS, measurements were performed at room temperature in PBS buffer using a Nanowizard III AFM (JPK Instruments) and oxide-sharpened micro-fabricated Si_3_Ni_4_ cantilevers with a nominal spring constant of ~0.01 N m^−1^ (MSCT) (Microlevers; Bruker Corporation). The spring constants of the cantilevers were measured using the thermal noise method. For the experiments, bacteria expressing chimeric SpsL A+SD were immobilized on polystyrene substrates. Adhesion maps were obtained by recording 16 x 16 force-distance curves on areas of 500 by 500 nm^2^ with an applied force of 250 pN, a constant approach and retraction speed of 1 m s^-1^, and a contact time of 0 ms. Adhesion force and rupture distance histograms were obtained by calculating the force and rupture distance of the last peak for each curve. Data were analyzed with the data processing software from JPK Instruments.

### Canine fibrinogen α-chain sequence analysis

Genomic DNA was isolated from whole canine blood using the method described previously [65]. The region of interest in the fibrinogen α-chain was amplified using Q5 Hot Start high-fidelity DNA polymerase (NEB) and purified using Monarch PCR & DNA Cleanup kit (NEB). Purified PCR products were analyzed by Sanger sequencing (Eurofins) and DNAStar SeqMan Pro 14 (Lasergene). Sequence alignment was performed using MegAlign (Lasergene) and PRALINE [66].

### Biofilm assay

Biofilm assays were performed using *S*. *pseudintermedius* strains expressing pALC2073 constructs of full length SpsL or A-domain+SD. Strains were grown in TSB supplemented with 0.5% (v/v) glucose and 3% (v/v) NaCl. 96-well tissue culture plates were coated overnight at 4°C with 100 nM bovine, canine, human, or ovine fibrinogen with some wells left uncoated. Overnight cultures were diluted to an OD_600nm_ of 0.05 and 100 μl applied to the plate and incubated at 37°C for 24 h. The plates were washed three times with PBS and the bacteria fixed with 25% (v/v) formaldehyde (Sigma) for 30 min. After washing, the plates were stained with 0.5% (v/v) crystal violet (Sigma) for 3 min and then solubilized with 5% acetic acid (v/v). Plates were analyzed using a Synergy HT plate reader (BioTek) at 595 nm wavelength.

### Neutrophil phagocytosis by flow cytometry

50 ml of venous blood was drawn from healthy volunteers and mixed with 6 ml of acid-citrate-dextran (Sigma). Human neutrophils were isolated as outlined previously [67] and suspended to a final concentration of 2.5 x 10^6^ cells ml^-1^ in RPMI-1640 (Gibco) containing 0.05% human serum albumin (Sigma). 2.5 x 10^6^ CFU of bacteria, previously labelled with FITC using a method previously described [68], were opsonized with 50 nM of extracellular matrix protein at 37°C for 15 min and diluted to 1 ml in RPMI-1640 containing 0.05% human serum albumin. 2.5 x 10^5^ CFU were then opsonized with 10% human serum in 2 ml 96-well v-bottomed plates (Corning) at 37°C for 15 min. 2.5 x 10^5^ neutrophils were added to the opsonized bacteria (MOI of 1) and incubated at 37°C for 15 min with shaking at 750 rpm. The samples were fixed with 1% (v/v) paraformaldehyde (Fisher Scientific) and incubated at 4°C for at least 30 min. Phagocytosis was measured in comparison to serum-only controls using the BD LSRFortessa X20 cell analyzer.

### Statistical analysis

Data is presented in Prism 6 (Graphpad) with statistical analysis performed using Minitab 16. All data was analyzed for normality, using the Anderson-Darling test, and equal variance before choosing the method of statistical analysis. Multiple comparisons were performed were appropriate. ELISA-type binding assays and bacterial adherence assays were analyzed at one protein concentration. For data displaying statistical significance, the following symbols are used, * p≤0.05, ** p≤0.01, and *** p≤0.001.

## Supporting information

S1 Fig Recombinant truncates of SpsL do not adhere to fibrinogen. (A) Schematic of the SpsL recombinant truncates generated in this study. Adherence of recombinant proteins purified from *E*. *coli* to (B) canine or (C) human fibrinogen coated at 20 μg ml^-1^. (D) Adherence of canine fibrinogen to SpsL recombinant truncates purified from *E*. *coli* and coated at 10 μM. (E) Adherence of recombinant proteins purified from *E*. *coli* to fibronectin coated at 10 μg ml^-1^. (F) Adherence of recombinant proteins purified from *S*. *pseudintermedius* supernatant to canine fibrinogen coated at 10 μg ml^-1^. Binding of recombinant proteins was detected using 0.1 μg ml^-1^ mouse anti-His IgG-HRP. (G) Adherence of *S*. *aureus* SH1000Δ*clfA*Δ*clfB*Δ*fnbA*Δfnb*B* expressing ClfB or SpsL to canine fibrinogen as quantified by crystal violet staining. All data points represent the mean ± SD (n = 9).
(TIF)

S2 Fig Generation of the SpsLΔlatch expression construct. (A) Structural model of the N2N3 subdomains of SpsL produced in Phyre^2^ based on pdb 1N67. The model was annotated in PyMol. Residues identified to be important for ClfA adherence to fibrinogen are colored in orange with the predicted binding motif colored yellow and the predicted latch region (^502^NSASGSG^508^) colored in blue. (B) Western blot analysis of cell wall-associated samples of ED99Δ*spsL*Δ*spsD* expressing full length SpsL or SpsLΔlatch with a predicted molecular weight of 103 kDa. Expression was detected using 1 μg ml^-1^ chicken anti-SpsL N2N3 IgY and 0.5 μg ml^-1^ F(ab’)2 rabbit anti-chicken IgG-HRP. The cross-reactive band present in all samples below 55 kDa is thought to be the *S*. *pseudintermedius* antibody-binding protein SpsQ.
(TIF)

S3 Fig Adherence of ClfB to the α-chain recombinant fragments and adherence of SpsL to multiple sites in the tandem repeat region. (A, B) Adherence of ClfB expressed in SH1000Δ*clfA*Δ*clfB*Δ*tnbA*Δ*fnbB* to human and canine fragments or chimeric proteins. (C) Schematic of the canine tandem repeat region of the fibrinogen α-chain. (D) Schematic of the α-chain fragments covering the tandem repeat region generated and purified from *E*. *coli*. (E) Adherence of SpsL expressed in ED99Δ*spsL*Δ*spsD* to canine α-chain fragments. All data points represent the mean ± SD (n = 9). (F) Schematic of the α-chain deletion constructs. (G) Adherence of SpsL expressed in ED99Δ*spsL*Δ*spsD* to the canine α-chain deletion constructs. All data points represent the mean with error bars representing SEM (n = 9).
(TIF)

S4 Fig Sequence analysis of the SpsL fibrinogen-binding sites. (A) Sequence alignment of region P283-E474 of the canine fibrinogen α-chain from 11 canine breeds. The heterozygous alleles contain abbreviations of French bulldog (FB) and Labrador retriever (LR). (B) Sequence analysis of the region S423-E474 of the fibrinogen α-chain from bovine, canine, human, and ovine hosts. Both alignments were generated using the online MultAlin tool [69].
(TIF)

S1 Table Strains and plasmids used in this study. (DOCX)

S2 Table Oligonucleotides used in this study. (DOCX)

S1 Methods Methodology only used in the supporting information. (DOCX)
