# Supplementary figures and images for: Host-specialized fibrinogen-binding by a bacterial surface protein promotes biofilm formation and innate immune evasion
Source: PLoS Pathog. 2019 Jun 19;15(6):e1007816. doi: 10.1371/journal.ppat.1007816 (PMC6602291; doi:10.1371/journal.ppat.1007816)

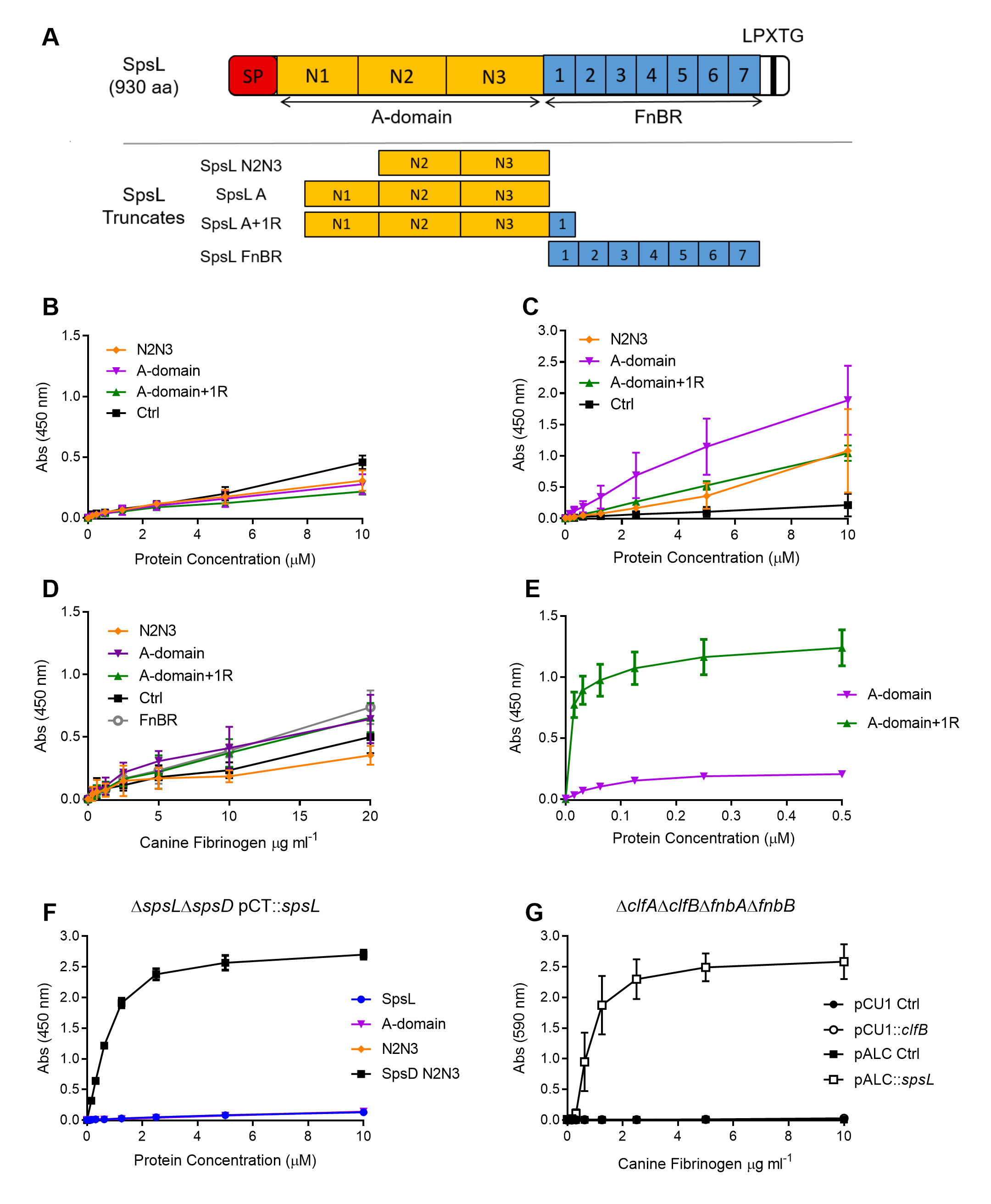

Supplement: S1 Fig — (A) Schematic of the SpsL recombinant truncates generated in this study. Adherence of recombinant proteins purified from E. coli to (B) canine or (C) human fibrinogen coated at 20 μg ml-1. (D) Adherence of canine fibrinogen to SpsL recombinant truncates purified from E. coli and coated at 10 μM. (E) Adherence of recombinant proteins purified from E. coli to fibronectin coated at 10 μg ml-1. (F) Adherence of recombinant proteins purified from S. pseudintermedius supernatant to canine fibrinogen coated at 10 μg ml-1. Binding of recombinant proteins was detected using 0.1 μg ml-1 mouse anti-His IgG-HRP. (G) Adherence of S. aureus SH1000ΔclfAΔclfBΔfnbAΔfnbB expressing ClfB or SpsL to canine fibrinogen as quantified by crystal violet staining. All data points represent the mean ± SD (n = 9). (TIF) [file ppat.1007816.s001.tif]

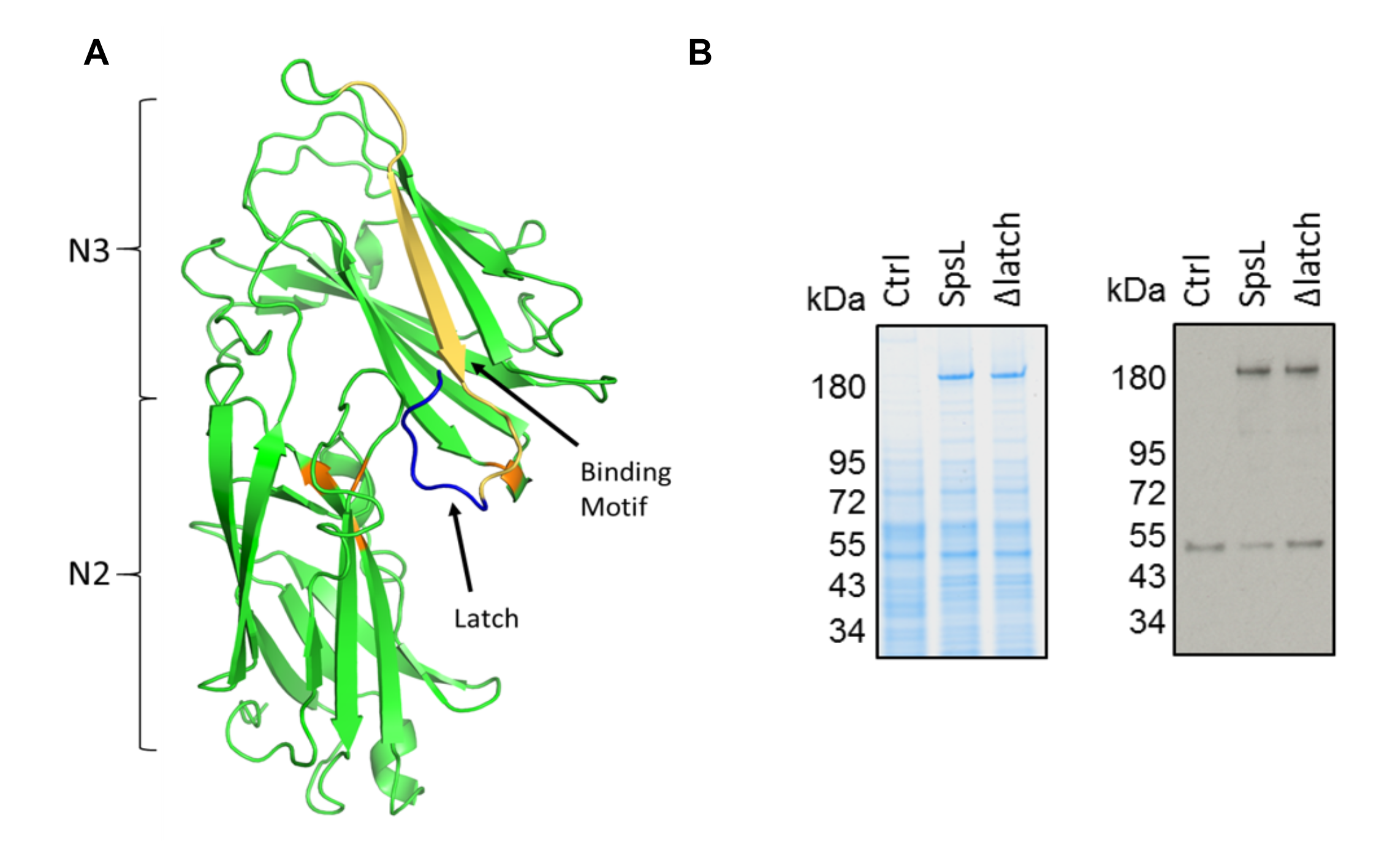

Supplement: S2 Fig — (A) Structural model of the N2N3 subdomains of SpsL produced in Phyre2 based on pdb 1N67. The model was annotated in PyMol. Residues identified to be important for ClfA adherence to fibrinogen are colored in orange with the predicted binding motif colored yellow and the predicted latch region (502NSASGSG508) colored in blue. (B) Western blot analysis of cell wall-associated samples of ED99ΔspsLΔspsD expressing full length SpsL or SpsLΔlatch with a predicted molecular weight of 103 kDa. Expression was detected using 1 μg ml-1 chicken anti-SpsL N2N3 IgY and 0.5 μg ml-1 F(ab’)2 rabbit anti-chicken IgG-HRP. The cross-reactive band present in all samples below 55 kDa is thought to be the S. pseudintermedius antibody-binding protein SpsQ. (TIF) [file ppat.1007816.s002.tif]

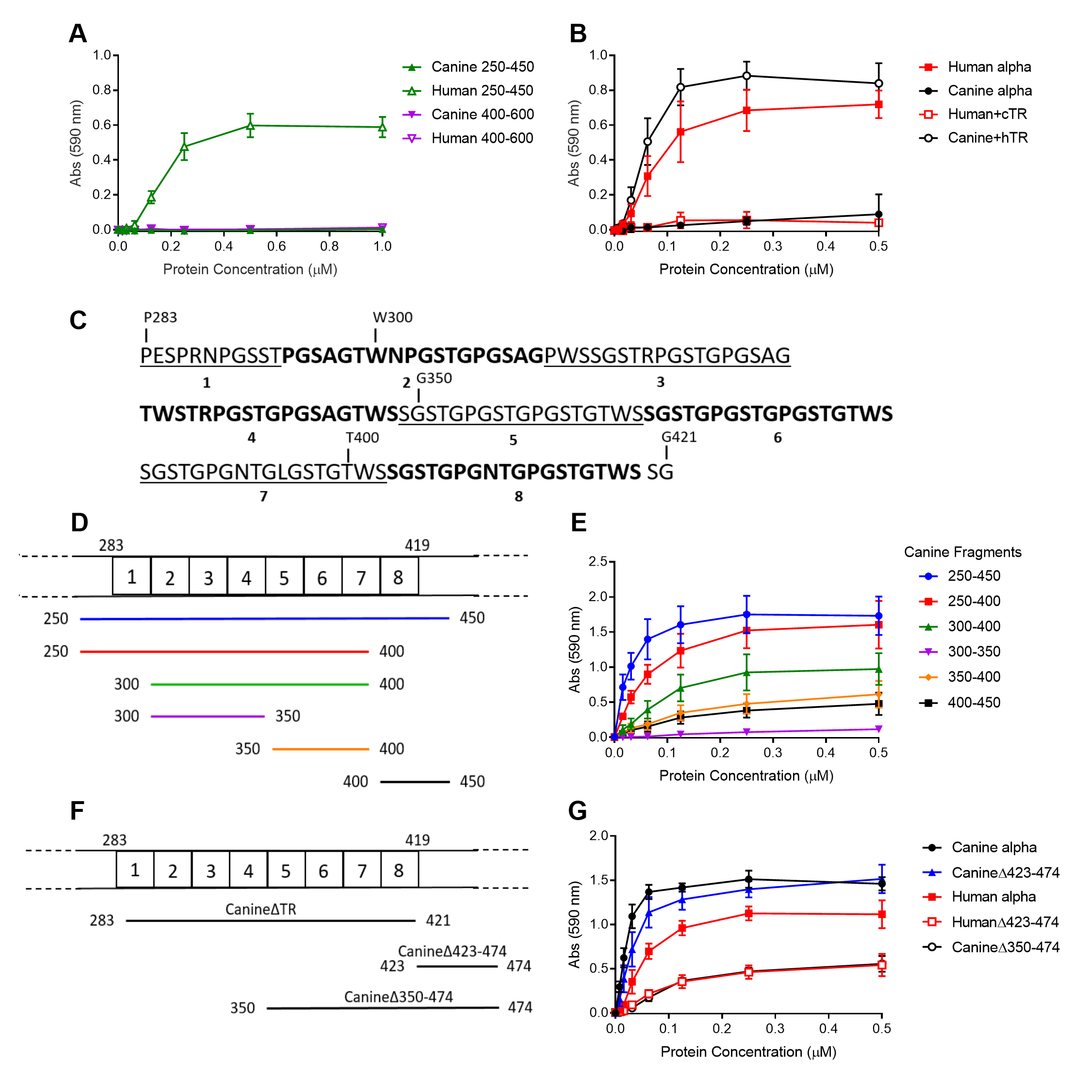

Supplement: S3 Fig — (A, B) Adherence of ClfB expressed in SH1000ΔclfAΔclfBΔtnbAΔfnbB to human and canine fragments or chimeric proteins. (C) Schematic of the canine tandem repeat region of the fibrinogen α-chain. (D) Schematic of the α-chain fragments covering the tandem repeat region generated and purified from E. coli. (E) Adherence of SpsL expressed in ED99ΔspsLΔspsD to canine α-chain fragments. All data points represent the mean ± SD (n = 9). (F) Schematic of the α-chain deletion constructs. (G) Adherence of SpsL expressed in ED99ΔspsLΔspsD to the canine α-chain deletion constructs. All data points represent the mean with error bars representing SEM (n = 9). (TIF) [file ppat.1007816.s003.tif]

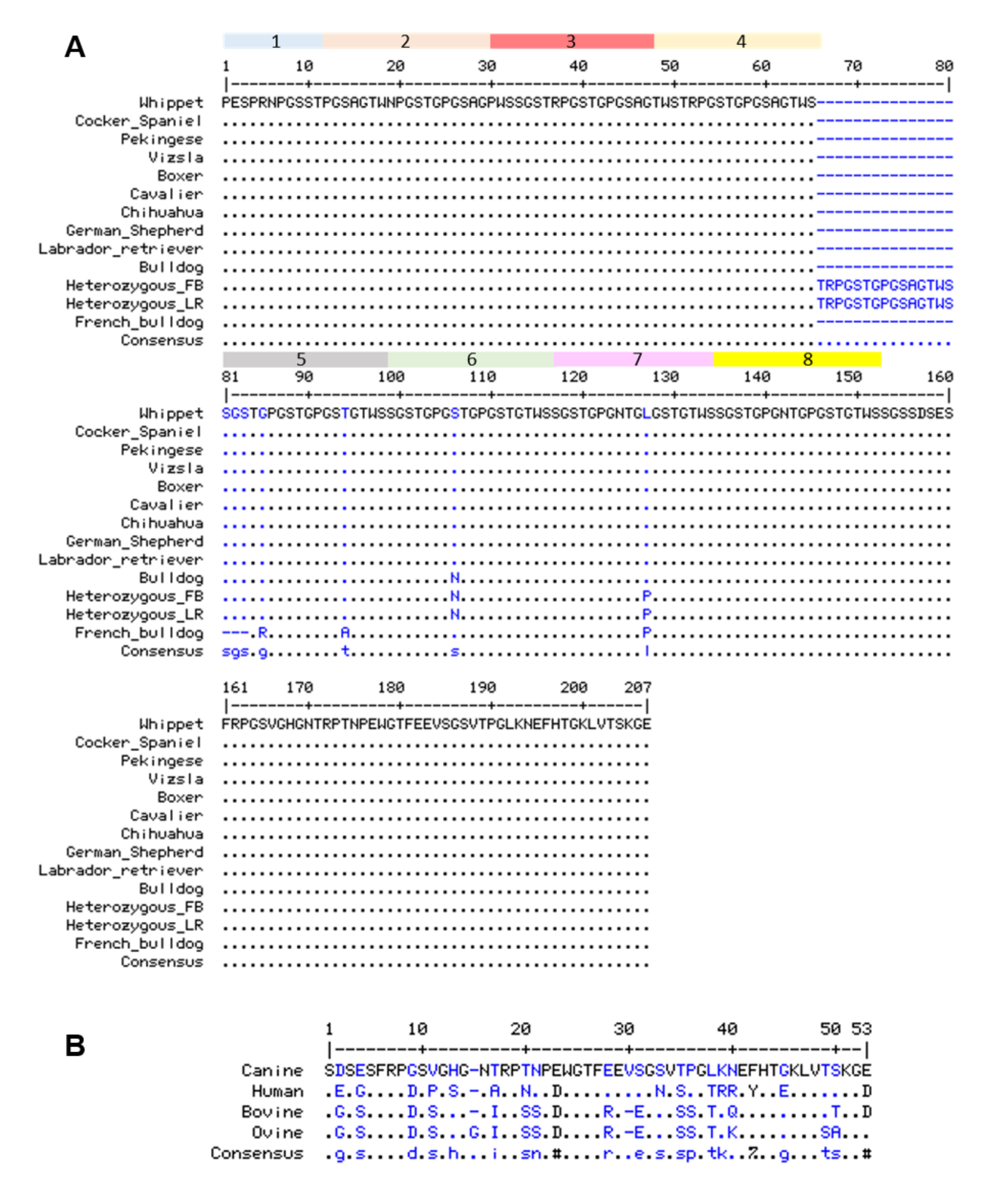

Supplement: S4 Fig — (A) Sequence alignment of region P283-E474 of the canine fibrinogen α-chain from 11 canine breeds. The heterozygous alleles contain abbreviations of French bulldog (FB) and Labrador retriever (LR). (B) Sequence analysis of the region S423-E474 of the fibrinogen α-chain from bovine, canine, human, and ovine hosts. Both alignments were generated using the online MultAlin tool [69]. (TIF) [file ppat.1007816.s004.tif]
