# Supplementary material for: Host-specialized fibrinogen-binding by a bacterial surface protein promotes biofilm formation and innate immune evasion
Source: PLoS Pathog. 2019 Jun 19;15(6):e1007816. doi: 10.1371/journal.ppat.1007816 (PMC6602291; doi:10.1371/journal.ppat.1007816)
Supplement: S1 Table — (DOCX) [file ppat.1007816.s005.docx]

S1 Table. Strains and plasmids used in this study.

| **Strain or plasmid** | **Genotype or description** | **Reference** |
| --- | --- | --- |
| *S. aureus* SH1000Δ*clfA*Δ*clfB*Δ*fnbA*Δ*fnbB* | Gene deletion of *clfA, clfB, fnbA, fnbB* | [1] |
| ***S. pseudintermedius*** |  |  |
| ED99 | Canine pyoderma isolate | [2] |
| ED99∆*spsD* | Gene deletion of *spsD* | [3] |
| ED99∆*spsL* | Gene deletion of *spsL* | [3] |
| ED99∆*spsL∆spsD* | Gene deletion of *spsL* and *spsD* | [3] |
| ED99∆*spsL* Rep | Repaired *spsL* gene | [4] |
| ***E. coli*** |  |  |
| Strataclone Solopack | *lacZ*∆*M15* mutation, *endA, recA-*deficient | Agilent |
| DC10B | DH10B background, Δ*dcm* | [5] |
| BL21 DE3 | T7 expression, Lon and OmpF protease-deficient | Invitrogen |
| DH5α | Cloning strain | Invitrogen |
| XL-1 Blue | *lacZ*Δ*M15, endA, recA*-deficient | Agilent |
| **Plasmids** |  |  |
| pCT | Derivative of pALC2073 engineered to generate in-frame fusions with an N-terminal 6xHis-tag and C-terminal Strep-tagII | O’Halloran DP and Geoghegan JA, unpublished |
| pCT*::spsL* | Secreted SpsL | This study |
| pCT*::spsL A* | Secreted SpsL A-domain | This study |
| pCT*::spsL N2N3* | Secreted SpsL N2N3 subdomains | This study |
| pALC2073 | Empty expression plasmid | [6] |
| pALC2073*::spsL* | Induced expression of SpsL | This study |
| pALC2073*::spsL* A-domain | Induced expression of SpsL A-domain | This study |
| pALC2073*::spsL* N2N3 | Induced expression of SpsL N2N3 subdomains | This study |
| pALC2073*::spsL* A+SD | Induced expression of SpsL A-domain plus ClfA SD repeats | This study |
| pALC2073*::spsL* N2N3+SD | Induced expression of SpsL N2N3 subdomains plus ClfA SD repeats | This study |
| pALC2073*::spsL* N1_21_+N2N3+SD | Induced expression of SpsL N2N3 subdomains plus N1 ^181^VSKEENTQVMQSPQDVEQHVG^201^ plus ClfA SD repeats | This study |
| pALC2073*::spsL*Δlatch | Induced expression of SpsL minus ^502^NSASGSG^508^ | This study |
| pALC2073*::spsL*+ClfB N2N3 | Induced expression of SpsL chimera containing ClfB N2N3 subdomains | This study |
| pALC2073*::spsL*+FnBPA N2N3 | Induced expression of SpsL chimera containing FnBPA N2N3 subdomains | This study |
| pCU1 | Empty expression plasmid | [1] |
| pCU1::*clfB* | Expression of ClfB with its native promoter | [7] |
| pT7::*spsD* N2N3 | His-tag SpsD N2N3 subdomains | This study |
| pT7::*spsL* N2N3 | His-tag SpsL N2N3 subdomains | This study |
| pQE30 | N-terminal 6xHis-tagged, T5 promoter | Qiagen |
| pQE-30::*spsL* A | His-tag SpsL A-domain | This study |
| pQE-30::*spsL* A+1R | His-tag SpsL A-domain + 1 repeat | This study |
| pQE-30::*spsL R* | His-tag SpsL Repeat domain | [3] |
| pQE-30::*spsD* FnBR | His-tag SpsD FnBR and B-domain | [2] |
| pQE-30:*α* | His-tag fibrinogen α-chain | [8] |
| pQE-30::*β* | His-tag fibrinogen β-chain | [8] |
| pQE-30::γ | His-tag fibrinogen γ-chain | [8] |
| pQE-30::canine α | synthesised DNA by IDT | This study |
| pQE-30::human α | synthesised DNA by IDT | This study |
| pQE-30::canine 20-150 | synthesised DNA by IDT | This study |
| pQE-30::canine 100-300 | synthesised DNA by IDT | This study |
| pQE-30::canine 250-450 | synthesised DNA by IDT | This study |
| pQE-30::canine 400-600 | synthesised DNA by IDT | This study |
| pQE-30::canine 550-750 | synthesised DNA by IDT | This study |
| pQE-30::canine 700-892 | synthesised DNA by IDT | This study |
| pQE-30::human 250-450 | His-tag human α fragment 250-450 | This study |
| pQE-30::human 400-600 | His-tag human α fragment 400-600 | This study |
| pQE-30::canine+hTR | His-tag canine α fragment with human tandem repeats | This study |
| pQE-30::human+cTR | His-tag human α fragment with canine tandem repeats | This study |
| pQE-30::canine 250-400 | His-tag canine α fragment 250-400 | This study |
| pQE-30::canine 300-400 | His-tag canine α fragment 300-400 | This study |
| pQE-30::canine 300-350 | His-tag canine α fragment 300-350 | This study |
| pQE-30::canine 350-400 | His-tag canine α fragment 350-400 | This study |
| pQE-30::canine 400-450 | His-tag canine α fragment 400-450 | This study |
| pQE-30::canineΔTR | His-tag canine α fragment with tandem repeat deletion | This study |
| pQE-30::canineΔ423-474 | His-tag canine α fragment with residues 423-474 deletion | This study |
| pQE-30::canineΔ350-474 | His-tag canine α fragment with residues 350-474 deletion | This study |
| pQE-30::humanΔ423-474 | His-tag human α fragment with residues 423-474 deletion | This study |

**References**

1. O'Neill E, Pozzi C, Houston P, Humphreys H, Robinson DA, Loughman A, et al. A novel *Staphylococcus aureus* biofilm phenotype mediated by the fibronectin-binding proteins, FnBPA and FnBPB. J Bacteriol. 2008;190(11):3835-50.

2. Simou C. Adherence of *Staphylococcus intermedius* to corneocytes of healthy and atopic dogs: effect of pyoderma, pruritus score, treatment and gender. Veterinary Dermatology. 2005;16(5):352-61.

3. Pietrocola G, Gianotti V, Richards A, Nobile G, Geoghegan JA, Rindi S, et al. Fibronectin binding proteins SpsD and SpsL both support invasion of canine epithelial cells by *Staphylococcus pseudintermedius*. Infect Immun. 2015;83(10):4093-102.

4. Richards AC, O'Shea M, Beard PM, Goncheva MI, Tuffs SW, Fitzgerald JR, et al. *Staphylococcus pseudintermedius* Surface Protein L (SpsL) Is Required for Abscess Formation in a Murine Cutaneous Infection Model. Infect Immun. 2018.

5. Monk IR, Shah IM, Xu M, Tan M-W, Foster TJ. Transforming the Untransformable: Application of Direct Transformation To Manipulate Genetically *Staphylococcus aureus* and *Staphylococcus epidermidis*. mBio. 2012;3(2):e002777.

6. Bateman BT, Donegan NP, Jarry TM, Palma M, Cheung AL. Evaluation of a Tetracycline-Inducible Promoter in *Staphylococcus aureus In Vitro* and *In Vivo* and Its Application in Demonstrating the Role of *sigB* in Microcolony Formation. Infect Immun. 2001;69(12):7851-7.

7. Ní Eidhin D, Perkins S, Francois P, Vaudaux P, Höök M, Foster TJ. Clumping factor B (ClfB), a new surface-located fibrinogen-binding adhesin of *Staphylococcus aureus*. Molecular Microbiology. 1998;30(2):245-57.

8. Vazquez V, Liang X, Horndahl JK, Ganesh VK, Smeds E, Foster TJ, et al. Fibrinogen is a ligand for the *S. aureus* MSCRAMM Bbp (Bone sialoprotein-binding protein). J Biol Chem. 2011;286(34):29797-805.
