# Supplementary material for: Host-specialized fibrinogen-binding by a bacterial surface protein promotes biofilm formation and innate immune evasion
Source: PLoS Pathog. 2019 Jun 19;15(6):e1007816. doi: 10.1371/journal.ppat.1007816 (PMC6602291; doi:10.1371/journal.ppat.1007816)
Supplement: S2 Table — (DOCX) [file ppat.1007816.s006.docx]

S2 Table. Oligonucleotides used in this study.

| **Primer Name** | **Sequence (5'-3'; restriction site underlined, complementary sites lowercase)** | **RE site** |
| --- | --- | --- |
| **SpsL expression constructs** | |  |
| *spsL A* F | GGATCCAATGAAGATGTCACTGAAACAA | *Bam*HI |
| *spsL A* R | CTGCAGCCATCCTATATCAATCACATGGC | *Pst*I |
| *spsL A+1R* F | GGATCCAATGAAGATGTCACTGAAACAA | *Bam*HI |
| *spsL A+1R* R | CTGCAGTGCTGTATCTTCTGTCATTTC | *Pst*I |
| *spsL* N2N3 F | GGATCCAACAGCGAAACTACTAT | *Bam*HI |
| *spsL* N2N3 R | GCGGCCGCAATATCTACTACATCA | *NotI* |
| *spsD* N2N3 F | GGATCCGAAAACGGAACAGATGTGACA | *Bam*HI |
| *spsD* N2N3 R | GCGGCCGCGGATAATCCGCATGAGTATCTTG | *NotI* |
| pQE-30 MCS F | CCCGAAAAGTGCCACCTG |  |
| pQE-30 MCS R | GTTCTGAGGTCATTACTGG |  |
| pCT F | GAATTCTGGTCTCATCCTC |  |
| pCT R | GAGCTCGGTACCATCAAG |  |
| pCT SpsL F | aataagcttgatggtaccgagctcTATGAGTTATTAGAAAGGGAGTTATTA |  |
| pCT SpsL R | aaattgaggatgagaccagaattcTTGCTGAGGTTGATTCTCTGTTACTTC |  |
| pCT SpsL A F | aataagcttgatggtaccgagctcTATGAGTTATTAGAAAGGGAGTTATTA |  |
| pCT SpsL A R | aaattgaggatgagaccagaattcTTCAATAATATCTACTACATCAGCTT |  |
| pCT SpsL N2N3 A | aataagcttgatggtaccgagctcTATGAGTTATTAGAAAGGGAGTTATTA |  |
| pCT SpsL N2N3 B | ATTCGCTTGAGCTTCATCATT |  |
| pCT SpsL N2N3 C | aatgatgaagctcaagcgaatGGTAAAGATATCTCTAATGAGGT |  |
| pCT SpsL N2N3 D | aaattgaggatgagaccagaattcTTGCTGAGGTTGATTCTCTGTTACTTC |  |
| **Complementation** | |  |
| pALC2073 spsL F | GGTACCGAAAGGGAGTTATTA | *Kpn*I |
| pALC2073 spsL R | GAGCTCTATTCTTTAATTCACCTTACTA | *Sac*I |
| pALC2073 SpsL A F | tttgggaaatgatgaagctcaagcgaatGAAGATGTCACTGAAACAACTG |  |
| pALC2073 SpsL A R | cactactactgtgttaacgtggttcttCACATGGCTATCTTCAATAATAT |  |
| pALC2073 F | AAGAACCACGTTAACACAGTAGTAGTG |  |
| pALC2073 R | ATTCGCTTGAGCTTCATCATTTCCCAAA |  |
| pALC2073 N2N3 F | tttgggaaatgatgaagctcaagcgaatGGTAAAGATATCTCTAATGAGG |  |
| pALC2073 N2N3 R | cactactactgtgttaacgtggttcttCACATGGCTATCTTCAATAATAT |  |
| pALC2073 A+SD F | tttgggaaatgatgaagctcaagcgaatGAAGATGTCACTGAAACAACTG |  |
| pALC2073 A+SD R | cactactactgtgttaacgtggttcttAGGTGAATTAGGCGGAACTA |  |
| pALC2073 N2N3+SD F | tttgggaaatgatgaagctcaagcgaatGGTAAAGATATCTCTAATGAGG |  |
| pALC2073 N2N3+SD R | cactactactgtgttaacgtggttcttAGGTGAATTAGGCGGAACTA |  |
| pALC2073 N1_21_+N2N3+SD F | tttgggaaatgatgaagctcaagcgaatGTGTCTAAAGAAGAAAATACTCAAGT |  |
| pALC2073 N1_21_+N2N3+SD R | cactactactgtgttaacgtggttcttAGGTGAATTAGGCGGAACTA |  |
| Latch F | tgaacggtgtgaagttttacgaaGAGTTGAAACCGAAACCACCTG |  |
| Lacth R | TTCGTAAAACTTCACACCGTTCA |  |
| pALC2073 MCS F | ATACCGCACAGATGCGTAAGG |  |
| pALC2073 MCS R | CGATGACTTAGTAAAGCACATCTAA |  |
| pALC2073 SpsL+ClfB N2N3 F | caagacgttgaacaacatgtaggtGCTGAACCGGTAGTAAATGCTGCT |  |
| pALC2073 SpsL+ClfB N2N3 R | tgtattccatcctatatcaatcacatgTGGAGTTGGGTCTTTCGGATTTAC |  |
| pALC2073 SpsL+FnBPA N2N3 F | caagacgttgaacaacatgtaggtACGGGTACAGATGTAACAAGTAAAGTT |  |
| pALC2073 SpsL+FnBPA N2N3 R | tgtattccatcctatatcaatcacatgTTCCTCTTCAACAGTAGTTACTAAATTCTT |  |
| pALC2073 SpsL N2N3 chimera F | CATGTGATTGATATAGGATGGAATACA |  |
| pALC2073 SpsL N2N3 chimera R | ACCTACATGTTGTTCAACGTCTTG |  |
| **Fibrinogen α-chain expression constructs** | | |
| h250-450 F | GGATCCGCACTGACCGATATG | *Bam*HI |
| h250-450 R | AAGCTTTTCTTCAAAGGTGCCCCAAT | *Hind*III |
| h400-600 F | GGATCCGGTCATTGGACCAGCGAAA | *Bam*HI |
| h400-600 R | AAGCTTATATGAGCTGCTTTTACCACGACTC | *Hind*III |
| canine α F | AGCGATAGCGAAAGTTTTCG |  |
| canine α R | AACGCTACCGGTGCCAT |  |
| human TR F | atggcaccggtagcgttACCGAAAGTCCGCGTAATC |  |
| human TR R | cgaaaactttcgctatcgctATGCCACTGACCGGTACTACCGCTAA |  |
| human α F | AGCGAAAGCGGTAGCTTTCGTCC |  |
| human α R | TTCGCTACCGGTGCCATAGCTGG |  |
| canine TR F | ccagctatggcaccggtagcgaaCCGGAAAGTCCGCGTAATCC |  |
| canine TR R | ggacgaaagctaccgctttcgctTGAACCGCTAGACCAAGTTCCG |  |
| c250-400 F | GGATCCGCACTGATGGAAATGAAA | *Bam*HI |
| c250-400 R | AAGCTTCGTACCGGTTGAGCCCAGAC | *Hind*III |
| c300-400 F | GGATCCTGGAATCCGGGTTCAAC | *Bam*HI |
| c300-400 R | AAGCTTCGTACCGGTTGAGCCCAGAC | *Hind*III |
| c300-350 F | GGATCCTGGAATCCGGGTTCAAC | *Bam*HI |
| c300-350 R | AAGCTTGCCACTGCTCCAAGTGCCAGC | *Hind*III |
| c350-400 F | GGATCCTCTACCGGACCAGGCTCTAC | *Bam*HI |
| c350-400 R | AAGCTTCGTACCGGTTGAGCCCAGAC | *Hind*III |
| c400-450 F | GGATCCTGGTCTAGTGGCTC | *Bam*HI |
| c400-450 R | AAGCTTTTCTTCAAAGGTGCCCCATTC | *Hind*III |
| canineΔTR F | atggcaccggtagcgttAGCGATAGCGAAAGTTTTCG |  |
| canineΔTR R | AACGCTACCGGTGCCAT |  |
| canineΔ423-474 F | ccggaacttggtctagcggttcaAAAGAACTGCTGATTGGCAATGAAAAAGT |  |
| canineΔ423-474 R | TGAACCGCTAGACCAAGTTCCGG |  |
| canineΔ350-474 F | gctggcacttggagcagtggcAAAGAACTGCTGATTGGCAATGAAAAAGT |  |
| canineΔ350-474 R | GCCACTGCTCCAAGTGCCAGC |  |
| humanΔ423-474 F | gttagcggtagtaccggtcagtggcatAAAGAACTGCGTACCGGTAAAGAAAAAGT |  |
| humanΔ423-474 R | ATGCCACTGACCGGTACTACCGCTAAC |  |

RE – restriction enzyme, F - forward primer, R – reverse primer, MCS – multiple cloning site
