# Supplementary material for: Host-specialized fibrinogen-binding by a bacterial surface protein promotes biofilm formation and innate immune evasion
Source: PLoS Pathog. 2019 Jun 19;15(6):e1007816. doi: 10.1371/journal.ppat.1007816 (PMC6602291; doi:10.1371/journal.ppat.1007816)
Supplement: S1 Methods — (DOCX) [file ppat.1007816.s007.docx]

**S1 Methods. Methodology only used in the supporting information.**

**Recombinant protein induction and purification**

Recombinant hexa-Histidine-tagged proteins expressed in *E. coli* were cultured to OD_600_ of 0.6 and induced using 1 mM IPTG at either 37°C for 4 h or 16°C overnight. Recombinant SpsL proteins were purified under native conditions (20 mM Tris, 500 mM NaCl, pH 8.0) using 1 ml pre-packed HisTrap FF crude IMAC columns (GE Healthcare) with a peristaltic pump P-1 (GE Healthcare) or using Ni-NTA Superflow resin (Qiagen) with the AKTA prime purification system (GE Healthcare). Bacterial lysis was performed in binding buffer containing 10 mM imidazole supplemented with cOmplete protease inhibitor (Roche) using a One-Shot cell disruptor (Constant Systems) with a single pulse at 25 kpsi. Lysates were pelleted at 16000 x *g* for 20 min and the supernatant filter sterilized. Elution was performed over a gradient to 100 mM imidazole. After analysis by 12 % SDS-PAGE, the protein elutions were concentrated using Amicon UItra-15 centrifugal filter units (Millipore) and dialyzed to PBS using Float-A-Lyzer tubing (Spectrum Labs). Protein quantification was performed using a NanoDrop ND1000 (Thermo).

Recombinant proteins purified from *S. pseudintermedius* were induced into the supernatant using 3 µg ml^-1^ anhydrotetracycline at 37°C for 5 h after culturing to an OD_600_ of 0.5. 436 g of ammonium sulfate was dried overnight at 120°C on a metal tray and then gradually applied to 1 L of filter-sterilized supernatant stirred in a 4 L flask. The sample was stirred for 3 h and then left to precipitate without stirring for 2 h. After centrifugation the pellet was suspended in native binding buffer and used for purification following the native purification protocol outlined above using a 5 ml pre-packed HisTrap FF crude IMAC columns (GE Healthcare) with a peristaltic pump P-1 (GE Healthcare). Protein was dialyzed as stated above and quantified using a Nanodrop 1000 (Thermo Scientific).

**ELISA-type binding assay**

Proteins were coated overnight in PBS at 4°C at a single concentration in 96-well MaxiSorp plates (Nunc). All incubation steps were performed at 37°C for 1 h. Wells were blocked with 8 % (w/v) milk-PBS before application of two-fold serial dilutions of recombinant or fibrinogen protein in PBS. After washing, antibodies were applied in 1 % (w/v) milk-PSBT (0.1 % (v/v) Tween-20) with detection of recombinant proteins performed using 0.1 µg ml^-1^ anti-poly-His IgG-HRP (Alpha Diagnostic International). Antibody detection of fibrinogen was performed using 0.17 µg ml^-1^ rabbit anti-canine fibrinogen IgG (Abcam) and 0.2 µg ml^-1^ goat anti-rabbit IgG-HRP (Abcam). Peroxidase activity was detected with supersensitive TMB liquid substrate (Sigma) and the reaction stopped with 0.16 M sulfuric acid. Absorbance was measured using a Synergy HT plate reader (BioTek) at 450 nm wavelength.

**Structural modelling of SpsL N2N3**

The sequence of SpsL A-domain was analyzed for structural modelling using Phyre^2^ [1]. The predicted structure was generated based on pdb 1N67, ClfA N2N3 [2]. The predicted structural model was analyzed and annotated in PyMol (The PyMOL Molecular Graphics System, Version 2.0 Schrӧdinger, LLC).

**References**

1. Kelley LA, Mezulis S, Yates CM, Wass MN, Sternberg MJE. The Phyre2 web portal for protein modeling, prediction and analysis. Nature Protocols. 2015;10(6):845-58.

2. Deivanayagam CCS, Wann ER, Chen W, Carson M, Rajashankar KR, Hook M, et al. A novel variant of the immunoglobulin fold in surface adhesins of *Staphylococcus aureus*: crystal structure of the fibrinogen-binding MSCRAMM, clumping factor A. Embo J. 2002;21(24):6660-72.
